# Gradient area-selective deposition for seamless gap-filling in 3D nanostructures through surface chemical reactivity control

**DOI:** 10.1038/s41467-022-35428-6

**Published:** 2022-12-09

**Authors:** Chi Thang Nguyen, Eun-Hyoung Cho, Bonwook Gu, Sunghee Lee, Hae-Sung Kim, Jeongwoo Park, Neung-Kyung Yu, Sangwoo Shin, Bonggeun Shong, Jeong Yub Lee, Han-Bo-Ram Lee

**Affiliations:** 1 Department of Materials Science and Engineering, Incheon National University, Incheon, 22012 Korea; 2 Beyond Silicon Lab, Samsung Advanced Institute of Technology, Gyeonggi, 16678 Korea; 3 Department of Chemical Engineering, Hongik University, Seoul, 04066 Korea; 4 Department of Mechanical and Aerospace Engineering, University at Buffalo, Buffalo, NY 14260 USA

**Keywords:** Synthesis and processing, Chemical engineering

## Abstract

The integration of bottom-up fabrication techniques and top-down methods can overcome current limits in nanofabrication. For such integration, we propose a gradient area-selective deposition using atomic layer deposition to overcome the inherent limitation of 3D nanofabrication and demonstrate the applicability of the proposed method toward large-scale production of materials. Cp(CH_3_)_5_Ti(OMe)_3_ is used as a molecular surface inhibitor to prevent the growth of TiO_2_ film in the next atomic layer deposition process. Cp(CH_3_)_5_Ti(OMe)_3_ adsorption was controlled gradually in a 3D nanoscale hole to achieve gradient TiO_2_ growth. This resulted in the formation of perfectly seamless TiO_2_ films with a high-aspect-ratio hole structure. The experimental results were consistent with theoretical calculations based on density functional theory, Monte Carlo simulation, and the Johnson-Mehl-Avrami-Kolmogorov model. Since the gradient area-selective deposition TiO_2_ film formation is based on the fundamentals of molecular chemical and physical behaviours, this approach can be applied to other material systems in atomic layer deposition.

## Abstract (web summary)

Integrating bottom-up and top-down fabrication techniques can overcome limits in nanofabrication. Here authors demonstrate an approach for area selective deposition using Ti as an inhibitor in the atomic layer deposition process to achieve controlled growth of seamless TiO_2_ layers on 3D structures.

## Introduction

Decades ago, top-down and bottom-up approaches were competitively studied for next-generation nanoscale fabrication. The top-down approach, which is primarily used for the nanofabrication of Si devices using photolithography and etching, has been recently used to fabricate 3D nanostructures, such as nanowires and nanodots, which were previously only obtainable through the bottom-up approach. The top-down approach also facilitates large-scale manufacturing, which has allowed this approach to dominate the current nanofabrication technology. Top-down-approach-based nanofabrication is essential for the construction of modern Si electronics and other devices, such as bio-medical and energy devices. In addition, many challenges that limit top-down-approach-based nanofabrication have been resolved by realising an integrated approach incorporating the bottom-up technology. An example of such an integrated approach is area-selective deposition (ASD) that is based on surface chemical reactivity control.

Atomic layer deposition (ALD) enables nanoscale precise thickness control, excellent uniformity and conformality because of the unique surface self-saturated reactions of precursors and counter-reactants^1–3^. Because ALD allows strong surface-dependent growth, a film could be selectively deposited on a specific area to achieve chemical reactivity control. Self-assembled monolayers, organic molecules, polymers and ALD precursors have been introduced to inhibit or promote the surface chemical reactivity of ASD-grown films^4–7^. An inhibitor selectively adsorbs on a specific surface and inhibits the subsequent ALD growth on the surface only, allowing bottom patterns to transfer to upper patterns through ASD. For example, in our previous study^8^, ASD was conducted using a Si precursor inhibitor that was previously used for the ALD of SiO_2_. The study highlighted that the Si precursor inhibitors selectively adsorbed on a OH-terminated SiO_2_ surface but not on a H-terminated Si surface, inhibiting the growth of Pt and Ru ALD. Until now, most ASD-based research has focused on the selective growth between two different surfaces such as SiO_2_/Si, SiO_2_/Cu and SiO_2_/HfO_2_ for expanding the application scope of ASD in material fabrication and on the adsorption selectivity of inhibitors^8–12^. If the growth selectivity in ASD can be controlled using other factors, such as the geometry of nanopatterns, but not by surface termination, then ASD can be extended to other bottom-up-approach-based applications to overcome the limits impeding nanofabrication.

**Fig. 1:**
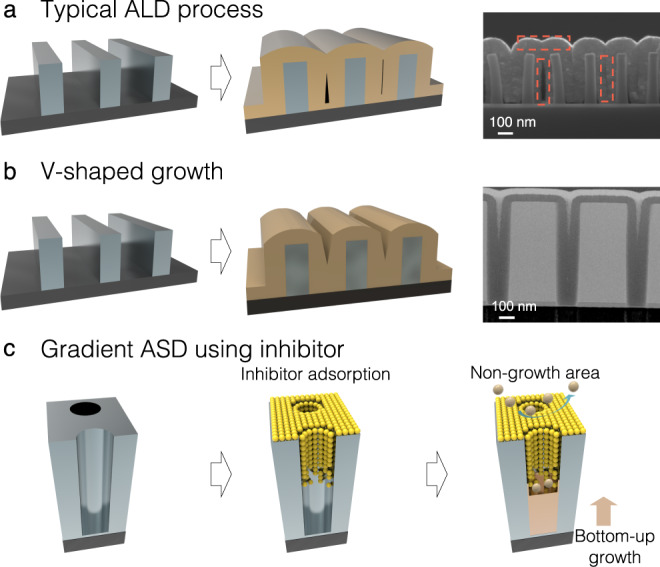
Schematic illustrations of ASD processes for seamless gap-filling. **a** Typical ALD process. **b** ALD process for V-shaped growth. **c** The proposed gradient ASD process using an inhibitor.

Isotropic growth is the main advantage of ALD; however, such growth induces the negative side effect of seam and void formation in high-aspect-ratio 3D structures such as holes and trenches^13^. As the number of ALD cycles increases, structure openings become closed (pinch-off) at a certain film thickness, forming voids or seams along the centreline as shown in Fig. 1a. The seams and voids are unavoidable critical defects formed during nanofabrication; they degrade device performance, electrical conductivity and thermal or mechanical properties^14–16^. To avoid seam formation, anisotropic growth inside the structure, like V-shaped growth, is required but cannot be achieved from the isotropic growth during ALD (Fig. 1b)^14^.

Seamless deposition of TiO_2_ in 3D nanostructures is essential for optical device applications, such as the fabrication of meta-surfaces^17,18^, but there is no proper deposition method to realize it^19,20^. In this study, we introduce a process of gradient ASD of a TiO_2_ film that allows seamless gap-filling; the process is illustrated in Fig. 1c. Through ALD, a TiO_2_ film is deposited on high-aspect-ratio (AR) hole patterns; a Ti inhibitor layer is then adsorbed onto this TiO_2_ film. The adsorption densities of the inhibitor vary depending on the geometrical positions and induce different blocking properties in the inhibitor. This causes ALD-grown TiO_2_ films to grow anisotropically, resulting in gap-filling without seam formation and realising the above-mentioned objective of the proposed gradient ASD concept. To inhibit TiO_2_ growth, herein, a Ti inhibitor is introduced, namely, trimethoxy-(pentamethylcyclopentadienyl)-titanium(IV) [TMPMCT], which was originally developed as a Ti precursor for ALD TiO_2_. TMPMCT-inhibited film growth and seamless gap-filling were investigated using various experimental and theoretical methods, including density functional theory (DFT) calculation, Monte Carlo (MC) simulation and the Johnson–Mehl–Avrami–Kolmogorov (JMAK) model. Initial experiments of gradient ASD were demonstrated in a lab-scale ALD system, and its wafer-scale demonstration was performed in a production-scale spatial-divided ALD system. Based on this research, we believe that the proposed approach can be applied for the ALD-assisted fabrication of other material systems.

## Results

**Fig. 2:**
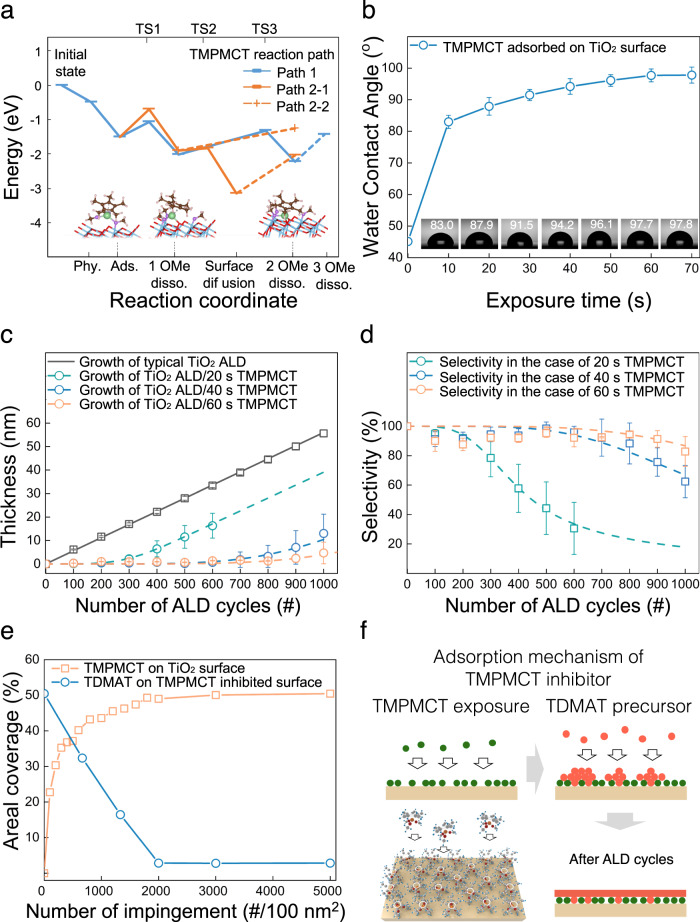
Adsorption behaviour and blocking property of TMPMCT inhibitor. **a** Surface reaction energy diagram of TMPMCT molecules on the TiO_2_ surface calculated by DFT. The structures below correspond to the Path 1. The dotted lines represent the reactions, where TS (transition states) structures were not calculated. Colours: Ti_surf_ (light blue), Ti_TMPMCT_ (yellow green), O_surf_ (red), O_TMPMCT_ (purple), C (brown), H (white pink). The corresponding chemical equations: Phy.: Cp(CH_3_)_5_Ti(OMe)_3_, Ads.: *-Cp(CH_3_)_5_Ti(OMe)_3_, 1OMe disso.: *-Cp(CH_3_)_5_Ti(OMe)_2_ + *-(OMe), 2OMe disso.: *-Cp(CH_3_)_5_Ti(OMe) + 2·*-(OMe), 3OMe disso.: *-Cp(CH_3_)_5_Ti + 3·*-(OMe). Phy. (Physisorption), Ads. (Adsorption), disso. (dissociation), surf (surface), * refers to adsorbed species. **b** WCA measurement with various exposure times of TMPMCT on the TiO_2_ surface. Fitting of the JMAK model (dashed lines) to the data (points with error bars) obtained from **c** the growth of subsequent TiO_2_ ALD on the TMPMCT inhibitor layer (20, 40 and 60 s), and **d** selectivity calculated from the thickness measured by ellipsometry. **e** Areal coverage of TMPMCT on the TiO_2_ surface (10 nm × 10 nm) using various impingement numbers calculated by MC simulation and the coverage of TDMAT on the TMPMCT-inhibited surface. **f** Adsorption mechanism of TMPMCT inhibitor. Unoccupied sites serve as starting points for the nucleation sites of TiO_2_ in subsequent ALD cycles. Source data are provided as a Source data file.

Previous reports suggest that TMPMCT is only oxidised by reactive oxidants, such as ozone and O_2_ plasma^21,22^, but not by H_2_O^23,24^. Thus, if TMPMCT covers a surface before an ALD process using a H_2_O counter-reactant, TMPMCT could inhibit the subsequent ALD growth by assuming no reaction between TMPMCT and the ALD precursor. This study used TMPMCT to inhibit subsequent TiO_2_ ALD using a tetrakis(dimethylamido)titanium (TDMAT) precursor and a H_2_O counter-reactant and investigated the adsorption stability of TMPMCT upon TDMAT and H_2_O exposure. In the DFT results in Fig. 2a, the molecular configuration of TMPMCT after adsorption indicates that the OMe ligands create bonds with the TiO_2_ surface and the Cp(CH_3_)_5_ ligand faces the outer surface. Adsorption of TMPMCT on the TiO_2_ surface is assumed to be dissociative adsorption via removal of individual OMe ligands as shown in Fig. 2a. A detailed description of the reaction mechanism is suggested in Supplementary Note 1. The adsorption of TMPMCT can be considered facile, as it is exothermic and does not involve significant activation energy. Then, when the surface becomes saturated by adsorbed TMPMCT, the outer surface would become terminated by the Cp(CH_3_)_5_ ligands. These cyclopentadienyl ligands with low chemical reactivity^24–26^ can then hinder the adsorption of subsequent TDMAT precursors.

Figure 2b shows the water contact angle (WCA) of the TMPMCT/TiO_2_ surface as a function of TMPMCT exposure time. While the WCA of the hydrophilic TiO_2_ surface^27–29^ was 45.1° before exposure to TMPMCT, it rapidly increased to 83.0° and 97.7° after 10 s and 60 s exposure of TMPMCT, respectively. The increase in WCA is attributed to the formation of a chemically inert surface resulting from the Cp(CH_3_)_5_Ti-ligand-terminated surface, as observed from the DFT results. No change of TiO_2_ thickness was observed after the reaction between TMPMCT and H_2_O (see Supplementary Fig. 1e), indicating TMPMCT is stable under H_2_O exposure. The blocking property of TMPMCT against 1000 cycles of ALD TiO_2_ was evaluated at different TMPMCT exposure times. Figure 2c shows that TiO_2_ growth on a TMPMCT-free surface is linear as a function of the ALD cycles. In contrast, there is almost no TiO_2_ film growth on the 20 s TMPMCT inhibition layer, the growth exhibits linearity as in the TMPMCT-free sample after 200 cycles. The TiO_2_ ALD film growth is inhibited for up to 700 and 900 cycles for the 40 s (blue points with error bars) and 60 s TMPMCT (orange points with error bars) inhibition layers, respectively. Then the TiO_2_ ALD film growth is linear as in the case of 20 s TMPMCT inhibition layer. The selectivity is calculated from the thickness measured by ellipsometry (Fig. 2c) and the results are shown in Fig. 2d. At small numbers of TiO_2_ ALD cycles, the deposit amount significantly affects the selectivity. In addition, ellipsometry has errors in the scale of a few angstroms, resulting in large deviations in the calculated selectivity for low-thickness films. However, separate SEM and TEM results (Supplementary Fig. 2) revealed that there was almost no growth of the TiO_2_ ALD thin film on 60 s TMPMCT/SiO_2_ (Supplementary Fig. 2a) and 60 s TMPMCT/10 nm TiO_2_/SiO_2_ (Supplementary Fig. 2b). Figure 2d indicates a very high calculated selectivity of >90% even after 900 cycles that is identical to the blocking of a 45-nm TiO_2_ thin film. The inhibition of the TiO_2_ film growth (using the same TDMAT precursor) herein was compared with that in the literature (Supplementary Table 1). The largest thickness of the inhibited TiO_2_ film was 50 nm, achieved using a 43 nm poly(methylmethacrylate) inhibition layer^30^, followed by 48 nm, achieved using a 76 nm polynorbornene inhibition layer^7^. Comparatively, a single inhibitor layer was sufficient to inhibit the TiO_2_ film growth to up to 45 nm in this study.

Once the TiO_2_ surface was exposed to TMPMCT, the OH-terminated TiO_2_ surface changed to a Cp(CH_3_)_5_Ti-terminated surface, and there was no significant reaction between the Cp(CH_3_)_5_Ti-terminated surface and Ti precursors, as expected based on the DFT calculation (see Supplementary Fig. 3). The maximum blocking cycle increased with increasing TMPMCT exposure time. Interestingly, the nucleation of subsequent TiO_2_ occurred over this specific cycle, leading to the degradation of the blocking property. Two possible reasons for this could be considered: (1) a direct reaction of the Cp(CH_3_)_5_Ti-terminated surface and Ti precursor over a larger number of cycles and (2) the nucleation of TiO_2_ on the defects in the TMPMCT inhibition layer. The DFT results eliminate the first reason. Therefore, we studied the nucleation of TiO_2_ on the inhibited layer using the theoretical simulations and experimental approaches described below.

Employing the previously-developed MC simulation, we investigated the physical adsorption and coverage of TMPMCT^8,12,31^ by only considering the physical steric hindrance of TMPMCT on a surface and using the adsorption configuration from the DFT calculation. Figure 2e depicts the areal coverages of TMPMCT on the TiO_2_ surface and TDMAT on the TMPMCT-inhibited surface. The area coverage of TMPMCT on the bare TiO_2_ surface rapidly increases with increasing TMPMCT impingement (orange line) and reaches a plateau of ~50%. Therefore, the coverage of subsequent TDMAT on the TMPMCT-covered surface gradually decreases (blue line). Although the coverage of TMPMCT on the TiO_2_ surface is saturated, some unoccupied adsorption sites remain. TMPMCT could not completely occupy the sites because of steric hindrance, and the TDMAT precursor can adsorb even on the TMPMCT-saturated surface (Supplementary Fig. 4). This result suggests that unoccupied sites, which are potential nucleation sites in the subsequent ALD process, exist even on the fully saturated TMPMCT layer. Therefore, the blocking property is degraded over specific cycles due to nucleation on unoccupied sites, as shown in Fig. 2f. This is consistent with our observations from Fig. 2c and d. In addition, an interesting finding is that the number of unoccupied sites changed with increased exposure time at the beginning, which may explain the different blocking properties of the Ti inhibitor witnessed during different exposure times.

Because TiO_2_ nucleates only on the defects of the inhibition layer, island growth occurs based on the Volmer–Weber island growth mode in the subsequent ALD process^32–34^. To understand the degradation process quantitatively, we adopted a kinetic model based on the Johnson–Mehl–Avrami–Kolmogorov (JMAK) model^4,35,36^ (details in Supplementary Note 2, Supplementary Fig. 5). Based on the best fit results, the parameters including $\,\dot{G}$, ${\dot{N}}_{0}$ and *v*_*d*_ are listed in Supplementary Table 1, and the fitting results with the experimental data are plotted in Fig. 2c and d (dashed lines). Figure 2c depicts an high goodness of fits for the thickness vs. ALD cycle number data of 0.933, 0.908 and 0.939 for 20 s, 40 s and 60 s of exposure time, respectively. Similarly, the selectivity data exhibits a highly consistent fit, as depicted in Fig. 2d. In addition, the nuclei density and nucleation growth of TiO_2_ on the inhibited surface by 20 s, 40 s and 60 s TMPMCT were simulated using the JMAK model (Supplementary Table 2 and Supplementary Fig. 6).

In fact, the defect of the inhibition layer is the OH-terminated original TiO_2_ surface; hence, ALD TiO_2_ easily nucleates on the defect sites during ALD if a defect site is still available. We assumed that the nucleation site density starts at 0 on the inhibition layer for JMAK simulation under ideal circumstances. Thus, the different nucleation site generation rates, ${\dot{N}}_{0}$ (presented in Supplementary Table 2) could be attributed to the different nucleation rates of TiO_2_ caused by the different defect densities in the TMPMCT inhibitor layer. The higher defect density caused by shorter inhibitor exposure results in a higher nucleation site density and reflects faster nucleation than that of lower defect density samples. In addition, the number of nucleation delay cycles resulting from the fitting are 80, 620 and 800 cycles for 20, 40 and 60 s, respectively. In an ideal ALD process, the precursor chemisorbs onto the potential sites or surface. If the surface species or termination is not highly reactive towards the precursor, it should be converted to a high-reactivity surface termination during the following reactant exposure time. The time required for low-to-high-reactivity conversion of the surface may affect the nucleation delay. For instance, H-terminated Si surface is not highly reactive toward the adsorption of tetrakis(dimethylamino)-hafnium (TDMAH) precursor; however, the surface can be converted to a OH-terminated Si surface via H_2_O counter reactant exposure or an O-terminated Si surface via O_3_ reactant pulsing, which exhibits high reactivity towards the TDMAH precursor^37^. In this study, this reason can be ruled out because the potential adsorption site for the next TDMAT precursor is the defect site, which is also the original TiO_2_ surface. When a TMPMCT inhibitor is used, the coverage of TMPMCT limits the potential adsorption site of the next TDMAT precursor, forming a sub-monolayer after each cycle. The time required for monolayer formation from a sub-monolayer can drive the nucleation delay. Thus, the slower nucleation rate observed on the longer-TMPMCT exposure sample leads to the longer nucleation delay, as simulated.

**Fig. 3:**
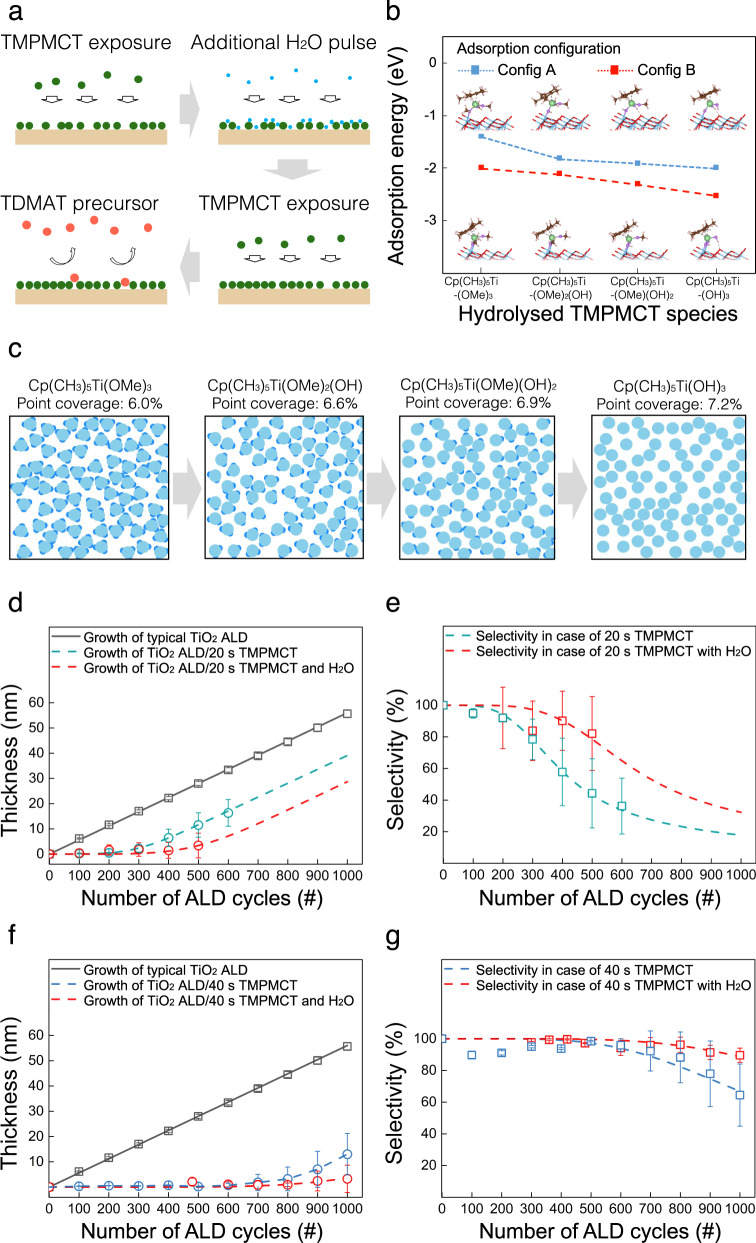
Blocking property of TMPMCT with an additional H_2_O pulse. **a** Schematic of TMPMCT exposure with an additional H_2_O pulse to improve coverage. **b** Adsorption energy of hydrolysed TMPMCT species, Cp(CH_3_)_5_Ti(OMe)_3-x_(OH)_x_ (x = 0, 1, 2 and 3), calculated by DFT. **c** MC simulation results for the adsorption of Cp(CH_3_)_5_Ti(OMe)_3-x_(OH)_x_ with steric hindrance in cases x = 1, 2 and 3. Fitting of the JMAK model to data (points with error bars) obtained from **d** growth and **e** selectivity in the case of 20 s TMPMCT with and without additional H_2_O pulse samples. Fitting of the JMAK model to data (points with error bars) obtained from **f** growth and **g** selectivity in the case of 40 s TMPMCT with and without additional H_2_O pulse samples. Source data are provided as a Source data file.

A theoretical interpretation of the experimental results suggests that the control of unoccupied sites, which are the same as defect sites, is the most important factor for a higher blocking property. The DFT results showed that the TMPMCT chemisorbs onto the TiO_2_ surface by releasing OMe ligands, but not all OMe ligands are dissociated. If the OMe ligand could also be eliminated, additional space would be available for the formation of the inhibitor, as depicted in Fig. 3a. Although the TMPMCT is not fully oxidised to TiO_2_ by an H_2_O reactant because of the remaining Cp(CH_3_)_5_ ligands, H_2_O can further eliminate the OMe ligands through a hydrolysis reaction with the adsorbed TMPMCT^38^. The DFT calculation modelled a reaction of H_2_O molecules with OMe ligands of the adsorbed TMPMCT and releases MeOH by-product, resulting in the formation of Cp(CH_3_)_5_Ti(OMe)_3-x_(OH)_x_ (x = 0, 1, 2 and 3). The energy for the ligand–exchange reaction between OMe and OH from H_2_O on the surface according to the number of H_2_O molecules is presented in Fig. 3b. Two adsorption configurations are considered due to a large degree of freedom, Config A and Config B. Both configurations show a decrease in E_ad_ as the more OMe ligands are modified into OH. From MC simulations for the adsorption of Cp(CH_3_)_5_Ti(OMe)_3-x_(OH)_x_ molecules, the results, shown in Fig. 3c, indicate that the point coverage of Cp(CH_3_)_5_Ti(OMe)_3-x_(OH)_x_ increased from 6.0% (without additional H_2_O and remaining 3OMe ligands) to 6.6%, 6.9% and 7.2% while eliminating 1OMe, 2OMe and 3OMe ligands, respectively. Thus, from the theoretical calculations, an additional H_2_O pulse can create more space for additional inhibitor adsorption, increasing the inhibitor coverage and decreasing the defect sites.

We modified the single 20 s TMPMCT pulse into two 10 s TMPMCT pulses disrupted by a 2.5 s pulse of H_2_O and the single 40 s TMPMCT pulse into two 20 s TMPMCT pulses disrupted by a 5 s pulse of H_2_O (Supplementary Fig. 7). We then evaluated their blocking properties up to 1000 cycles of ALD, as shown in Fig. 3d–g. As shown in Fig. 2, the TiO_2_ growth was inhibited for up to 200 and 700 cycles under 20 s and 40 s TMPMCT exposure conditions, respectively. With additional H_2_O exposure, however, the TiO_2_ growth was inhibited for up to 400 and 900 cycles for 20 s and 40 s samples, respectively, as shown in Fig. 3d–g. The fitting results with the JMAK model showed high goodness of fits as shown in Fig. 3d–g. From the parameters obtained from the fittings (Supplementary Table 3), the decrease in the nucleation site generation rate, ${\dot{N}}_{0}$, and the increase in the nucleation delays, *v*_*d*_, reflect an increase in the TMPMCT coverage by addition H_2_O, which leads to the reduction of defect sites, as predicted by DFT calculations and MC simulations.

**Fig. 4:**
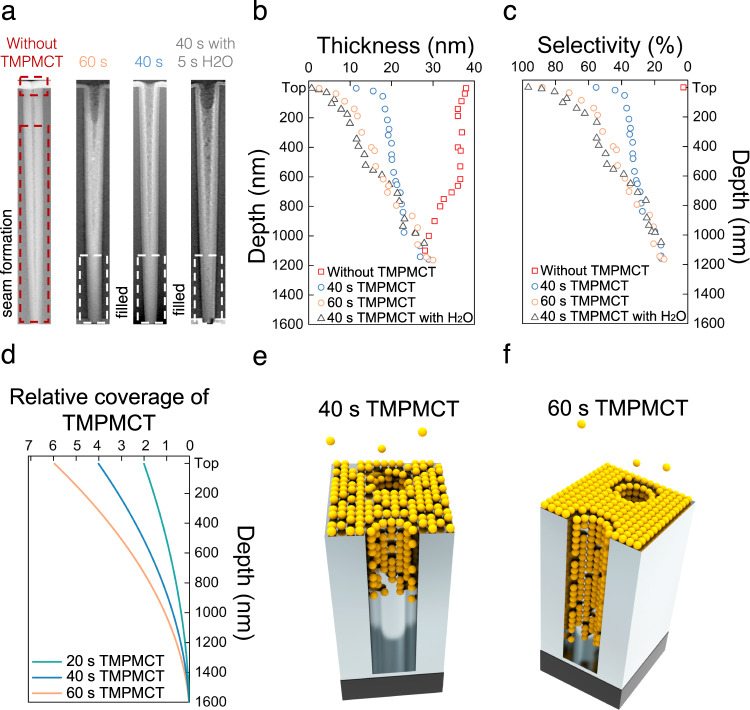
Hole pattern with a depth of 1600 nm, an opening diameter of 100 nm and a bottom diameter of 50 nm used for gap-filling. **a** TEM images of the subsequent 40 nm thick TiO_2_ ALD film on the 0, 40 and 60 s TMPMCT inhibitors and 40 s TMPMCT with an additional H_2_O/10 nm TiO_2_/SiO_2_ hole. **b** Growth and **c** selectivity calculated from the TEM results. **d** Relative coverage of various TMPMCT pulses, as calculated from the equation described in Supplementary Note 3 and depicted in illustrations **e** 40 s TMPMCT pulse, **f** 60 s TMPMCT pulse. Source data are provided as a Source data file.

From the results, we investigated different inhibitor coverages in a deep nanoscale hole pattern. We performed experiments on 8-inch wafers in a spatially divided ALD system, which has a high-volume manufacturing capability (Supplementary Fig. 8). We used 730 cycles of ALD on this nanoscale hole pattern to form a 40 nm thick TiO_2_ layer, based on the growth per cycle (GPC) of TiO_2_. As shown in Fig. 4a, while the hole deposited by TiO_2_ without TMPMCT almost was closed off with the formation of a seam, no pinch-off was observed for 40 s and 60 s TMPMCT exposure samples. The gradient blocking property was consistent for other sampling points in the same 8-inch wafer (TEM images in Supplementary Fig. 9). The thicknesses of TiO_2_ on the 40 s sample were distributed from 10 nm to 30 nm (blue points in Fig. 4b). The thicknesses of the 60 s sample were distributed more widely, from 2 nm to 30 nm with changing depth (orange points in Fig. 4b). In addition, the 40 s TMPMCT with H_2_O samples exhibited a thickness distribution similar to that of the 60 s TMPMCT sample without H_2_O. Near the opening, the blocking property of the longer exposure sample was more significant than that of the shorter exposure sample, similar to those on planar samples. The selectivity (Fig. 4c) of the 60 s sample was almost 90% at the top of the hole but decreased to 20% with increasing depth. The two 40 s samples with and without H_2_O also exhibited selectivity changes.

Because 3D nanostructures possess a much larger surface area than 2D planar surfaces, the adsorption behaviour on both structures should be different under the same exposure conditions, leading to different molecular adsorption densities. To quantify the results, we adopted a kinetic model to estimate the adsorption density of TMPMCT inside 3D nanoscale holes. The time *t* (s), which is required to cover the surface area of a 3D hole down to the depth *λ* (nm) can be calculated by the following equation (details in Supplementary Note 3)^39^:

$$t={\int }_{0}^{t}{{{{{\rm{d}}}}}}t=\frac{{{{{{\rm{Q}}}}}}\sqrt{2\pi {mkT}}}{P}\frac{p}{{{{{{{\rm{A}}}}}}}_{{{{{{\rm{hole}}}}}}}}{\int }_{0}^{L}{{{{{\rm{d}}}}}}\lambda \left(1+\frac{3p\lambda }{{16{{{{{\rm{A}}}}}}}_{{{{{{\rm{hole}}}}}}}}\right)$$

Although a perfectly cylinder-shaped hole was not used for this experiment, it can provide a good explanation for the approximate adsorption density changes. Figure 4d shows a plot of relative coverage of TMPMCT with different pulses versus the hole depth, calculated from the above-mentioned equation. The molecular adsorption on the top area is faster than that inside the hole because more time is required for molecules to reach the lower portion of the hole (Fig. 4e, f). Therefore, the relative coverage of TMPMCT was the highest at the top (depth = 0 nm) and decreased with increasing depth for all TMPMCT pulses. The longest TMPMCT pulse, 60 s, resulted in higher relative coverage at the top than the 40 s and 20 s TMPMCT pulses. As a result, the application of a short TMPMCT pulse would narrow the relative-coverage distribution. Therefore, more exposure time is required to cover a surface deeper inside the hole because of the geometrically limited flux of the inhibitor molecule. In other words, under constant exposure conditions, the adsorption density of TMPMCT should change with the changing depth, as plotted. Because the adsorption density of TMPMCT is proportional to the blocking property and selectivity, the relative coverage as a function of depth should be similar to the selectivity as a function of depth. Compared with Fig. 4c, plots resulting from the kinetic model demonstrate a high consistency, indicating that the blocking property changes depending on geometrical factors.

**Fig. 5:**
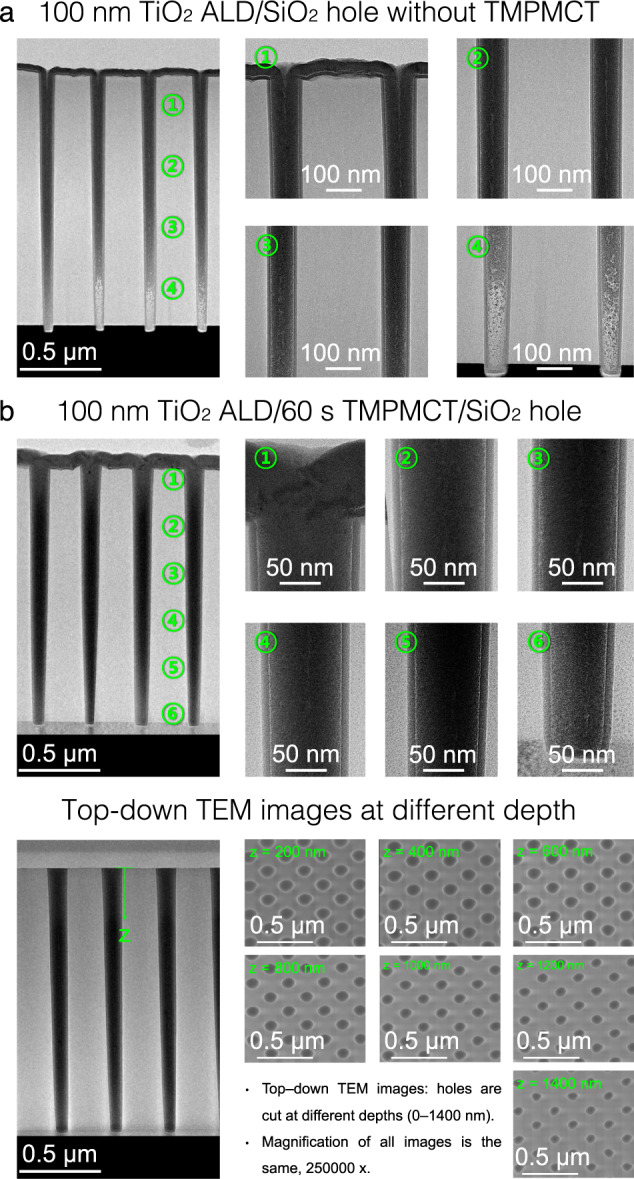
100 nm TiO_2_ ALD deposited in holes with and without TMPMCT inhibitor. **a** Cross-sectional TEM images of the hole pattern (depth = 1600 nm) for 100 nm TiO_2_ ALD without TMPMCT. Unfilled zones are observed at the bottom (zone 4), and seam formation occurs along the centreline of the hole (zones 2 and 3). **b** Cross-sectional TEM images of the 100 nm TiO_2_ ALD/60 s TMPMCT/SiO_2_ hole and top-down TEM images at different depths, demonstrating hole filling without any seam formation.

Based on this interpretation, we also performed a gradient ASD for seamless deposition on 8-inch wafers in a production-scale system (Supplementary Fig. 8). Figure 5a indicates that it is not feasible to fill the high-aspect-ratio hole (depth = 1600 nm) perfectly using 100 nm TiO_2_ ALD without TMPMCT. Cross-sectional TEM images show an unfilled zone and seam formation along the centreline of the hole. In addition, the seam formation significantly varied with the position in the wafer; thus, large voids were observed in the non-uniform regions of the wafer (TEM image in Supplementary Fig. 10 and uniformity data in Supplementary Table 4). Furthermore, when 60 s TMPMCT was used, perfect filling was observed without any seam or void formation, as shown in the cross-sectional and top-down TEM images acquired at different depths (Fig. 5b). Energy-dispersive spectroscopy (EDS) mapping (Supplementary Fig. 11) revealed that only Si, Ti and O were present without any significant amount of C. In addition, the results of the EDS line profile across the ASD TiO_2_/TMPMCT/SiO_2_ hole sample (Supplementary Fig. 12) demonstrate that no significant carbon impurities were left by TMPMCT between the substrate and TiO_2_, even after the cessation of the blocking property (Supplementary Fig. 12b). TMPMCT should exist in a Cp(CH_3_)_5_Ti form after ASD TiO_2_ (DFT results in Fig. 2a), forming Ti–C impurities; however, only a negligible amount of C was detected within the limits of the measurement (Supplementary Fig. 12d).

## Discussion

We proposed a Ti precursor inhibitor, TMPMCT, and studied its chemical and physical adsorption behaviour by using theoretical methods, DFT and MC simulations. The degradation of the TiO_2_ blocking property with an increasing number of ALD cycles was investigated by adopting an MC simulation and a kinetic growth model, the JMAK equation. It was revealed that the unoccupied sites in the inhibitor layer play an important role in the degradation of ASD TiO_2_ films, and the blocking property could be controlled by adjusting the surface coverage of the inhibitor. To improve the blocking property, an additional H_2_O pulse was added, as suggested by theoretical calculations and evaluation by DFT and MC simulation. H_2_O was found to eliminate the OMe ligands of the adsorbed TMPMCT, creating more space for additional adsorption of TMPMCT. A monolayer of the TMPMCT inhibitor was found to block a 45 nm thick TiO_2_ thin film in the subsequent ALD process, which is the thickest film thickness ever reported to date. In addition, the TMPMCT inhibitor with an H_2_O pulse demonstrated a significantly improved blocking property up to 49 nm of TiO_2_ blocking.

In 3D nanoscale hole patterns, ASD TiO_2_ films exhibited a geometrically anisotropic growth. By adopting a kinetic model, the adsorption behaviour of a TMPMCT inhibitor inside 3D holes was interpreted. The adsorption density of the inhibitor was found to vary inside the 3D holes because of the geometrically limited flux, leading to a gradient ASD. A perfectly seamless TiO_2_ deposition was obtained in the nanoscale 3D holes through ASD. Because the gradient ASD process works based on the fundamentals of molecular chemical and physical behaviours, this approach can be applied to other material systems in ALD. In addition, the experiments were repeated many times with a large number of samples on a production-scale system. The results were used for plotting graphs; parameters such as thickness and selectivity were averaged from different runs, and their standard deviations were determined (Supplementary Table 4 and Supplementary Fig. 14). The gradient ASD approach proposed herein has high reliability because it can be reproduced and applied to a large-scale ALD system. Hence, the fundamental understandings derived from this study can be directly applicable at manufacturing scales.

## Methods

### Sample preparation

Experiments were conducted on a lab-scale ALD machine (Atomic-Shell, CN1 Co., Korea) and scaled to a high volume production by a spatial-divided ALD (SALD) machine at the Samsung Advanced Institute of Technology. The SALD system includes an 8-inch rotary chamber that features a 16-split shower head with individual nozzle control. The susceptor rotates at 40 rpm, and the chamber can load three 8-inch wafers simultaneously, as shown in Supplementary Fig. 8. In the lab-scale ALD system, a small coupon wafer was loaded, and the precursor and counter-reactant were dosed on to the wafer via a nozzle line. Additional information on the lab-scale ALD system can be found in refs. 12, 40, 41.

The TiO_2_ precursors, titanium isopropoxide (TTIP) (Sigma-Aldrich) and tetrakis-(dimethylamido)titanium (TDMAT) (Oceanbridge Co., Ltd, Korea), were contained in a canister at 50 °C. The H_2_O reactant was kept at 25 °C. Trimethoxy-(pentamethylcyclopentadienyl)titanium(IV) (Cp(CH_3_)_5_Ti(OMe)_3_), abbreviated as TMPMCT (Ichems Co., Korea), was used as an inhibitor and was investigated at different temperatures. The substrates used in these experiments included a planar SiO_2_ wafer and hole patterns (1600 nm depth, 100 nm diameter opening, 50 nm bottom diameter). They were sequentially cleaned using acetone, isopropyl alcohol and deionised water and dried using N_2_ flow.

All experiments were performed at 180 °C^42–46^. The GPCs were approximately 0.40 and 0.55 Å/cycle for TTIP and TDMAT precursors, respectively (Supplementary Fig. 1a–d). The adsorption mechanism of the TMPMCT inhibitor on the TiO_2_ surface was investigated at different adsorption times (20–60 s) and canister temperatures (70–100 °C) (Supplementary Fig. 1e and f). The adsorption mechanism of the TMPMCT inhibitor on various substrates, namely, TiO_2_-deposited SiO_2_, Ru-deposited SiO_2_, HfO_2_-deposited SiO_2_, TiN and SiO_2_, was also investigated; the results are shown in Supplementary Fig. 1g. The TMPMCT inhibitor layer was used to evaluate the blocking property against TiO_2_ ALD on SiO_2_ and 10 nm TiO_2_-deposited SiO_2_ substrates, as shown in Supplementary Fig. 2. The results show that the TiO_2_ ALD thin films were obtained on the bare surfaces, but no TiO_2_ growth was observed on the TMPMCT-inhibited surfaces. The blocking property of the TMPMCT was examined at 20, 40 and 60 s exposure on TiO_2_-deposited SiO_2_ substrate, against 1000 cycles of TiO_2_ ALD. An additional H_2_O pulse was included to improve the coverage and blocking property of TMPMCT by inserting an H_2_O pulse to interrupt the continuous TMPMCT pulse. The sequence followed was TMPMCT pulse–additional H_2_O pulse–TMPMCT. In the gradient ASD experiments, we used an SiO_2_ hole pattern with a 1600 nm depth, 100 nm opening diameter and 50 nm bottom diameter. Before TMPMCT exposure, a 10 nm TiO_2_ was deposited on the SiO_2_ hole patterns to produce a TiO_2_ surface; hence, the opening diameter was reduced from 100 nm to 80 nm. We then investigated 20, 40 and 60 s of TMPMCT exposure and 40 s of TMPMCT with H_2_O pulses in the gap-filling processing of 40 nm and 100 nm TiO_2_.

### Sample characterization

The thickness of the deposited films was measured using field-emission scanning electron microscopy (FE-SEM; JEOL JSM-7001F; JEOL, Ltd.), which included ellipsometry at an incidence angle of 64.885° for thicknesses between 245 and 1000 nm (i.e., from −1.24 to 5.06 eV), and high-resolution transmission electron microscopy (JEM-2100F, JEOL) equipped with an energy-dispersive X-ray spectroscopy and a 50 nm^2^ probe (solid angle = 0.28 sr, take-off angle = 24.1°), as shown in Figs. 4, 5, and Supplementary Figs. 9, 10, 11, 12.

### Theoretical calculations

DFT calculations were performed using the Vienna ab initio simulation package version 5.4.4^47^. Within the DFT + U framework, the Perdew–Burke-Ernzerhof generalised gradient approximation functional was employed to treat the exchange-correlation energy^48^ while applying the D3(BJ) dispersion correction^49^. Using Dudarev’s approximation^50^, parameter U_eff_ (=U − J) for Ti was set to 3, which is an optimised value from the literature^51^. A clean anatase TiO_2_ (101) surface was used to simulate surface reactions. ALD-grown TiO_2_ was reported to exist in the anatase phase at a considered temperature in this study^41,52^, and the lowest surface energy was in the (101) plane^53^. Most H_2_O and OH moieties desorb from the anatase TiO_2_(101) surface at temperatures above 400 K^54^. The first surface model, a three-layered TiO_2_ surface with dimensions of 10.5 × 11.4 × 28 Å, was used to locate local or global minimum structures while the forces acting on the atoms were less than 0.02 eV Å^−1^. Energy barriers were obtained via the nudged elastic band method with a force tolerance of 0.05 eV Å^−1^ ^55,56^. The Cp(CH_3_)_5_Ti-terminated surface was simulated using the second surface model, two-layered TiO_2_ surface (20.9 × 11.4 × 28 Å), with a force tolerance of 0.02 eV Å^−1^. The surface models contained more than 10 Å of the vacuum region. All calculations were conducted with an energy cut-off level of 500 eV. A 2 × 2 × 1 k-point grid for the surface models was set according to the Monkhorst–Pack scheme after convergence test. The adsorption energy (E_ad_) was defined as E_ad_ = E_total_ − (E_slab_ + E_molecule_), where E_total_ and E_slab_ are the total electronic energies of the slab with and without an adsorbate, respectively, and E_molecule_ is the energy of the gaseous molecule.

The physical adsorption, the coverage of TMPMCT on the TiO_2_ surface, and the elimination of OMe ligands of TMPMCT using H_2_O were investigated via MC simulations. The MC simulations were performed using a modified algorithm, which was run on the simulation programme “R” (version 3.6.1; GUI 1.70; EL Capitan build 7684). Further information can be found in our previous reports^8,12^ (algorithm in Supplementary Fig. 13). We also employed the Johnson–Mehl–Avrami–Kolmogorov (JMAK) model to quantify the blocking property and degradation with experimental data^4,35,36^. The JMAK model was calculated using the Matlab software (Matlab R2018b version 9.5). In addition, the adsorption density of TMPMCT inside a 3D nanoscale hole was calculated using various TMPMCT pulses of 20, 40 and 60 s.

## Supplementary information

Supplementary Information

## Data Availability

The data that support the findings of this study are available from the corresponding authors upon reasonable request. Source data are provided with this paper.

## Source data

Source Data

## References

[1] Johnson RW, Hultqvist A, Bent SF (2014). A brief review of atomic layer deposition: from fundamentals to applications. Mater. Today.

[2] Cremers V, Puurunen RL, Dendooven J (2019). Conformality in atomic layer deposition: current status overview of analysis and modelling. Appl. Phys. Rev..

[3] Kim H, Lee H-B-R, Maeng W-J (2009). Applications of atomic layer deposition to nanofabrication and emerging nanodevices. Thin Solid Films.

[4] Lee H-B-R, Mullings MN, Jiang X, Clemens BM, Bent SF (2012). Nucleation-controlled growth of nanoparticles by atomic layer deposition. Chem. Mater..

[5] Kim YR (2022). Fluorine-containing polymeric inhibitor for highly selective and durable area-selective atomic layer deposition. Appl. Surf. Sci..

[6] Kim W-H (2016). A process for topographically selective deposition on 3D nanostructures by ion implantation. ACS Nano.

[7] Pattison TG (2020). Surface initiated polymer thin films for the area selective deposition and etching of metal oxides. ACS Nano.

[8] Khan R (2018). Area-selective atomic layer deposition using Si precursors as inhibitors. Chem. Mater..

[9] Mameli A (2017). Area-selective atomic layer deposition of SiO_2_ using acetylacetone as a chemoselective inhibitor in an ABC-type cycle. ACS Nano.

[10] Mackus AJM, Merkx MJM, Kessels WMM (2019). From the bottom-up: toward area-selective atomic layer deposition with high selectivity. Chem. Mater..

[11] Minaye Hashemi FS, Prasittichai C, Bent SF (2015). Self-correcting process for high quality patterning by atomic layer deposition. ACS Nano.

[12] Kim HG (2020). Effects of Al precursors on deposition selectivity of atomic layer deposition of Al_2_O_3_ using ethanethiol inhibitor. Chem. Mater..

[13] Talukdar TK, Girolami GS, Abelson JR (2019). Seamless fill of deep trenches by chemical vapor deposition: use of a molecular growth inhibitor to eliminate pinch-off. J. Vac. Sci. Technol. A.

[14] Talukdar TK, Wang WB, Girolami GS, Abelson JR (2018). Superconformal coating and filling of deep trenches by chemical vapor deposition with forward-directed fluxes. J. Vac. Sci. Technol. A.

[15] Abdolvand R, Johari H, Ho GK, Erbil A, Ayazi F (2006). Quality factor in trench-refilled polysilicon beam resonators. J. Microelectromechanical Syst..

[16] Schenk H, Dürr P, Kunze D, Lakner H, Kück H (2001). A resonantly excited 2D-micro-scanning-mirror with large deflection. Sens. Actuators A: Phys..

[17] Sun M (2019). Efficient visible light modulation based on electrically tunable all dielectric metasurfaces embedded in thin-layer nematic liquid crystals. Sci. Rep..

[18] Kim W (2022). Thermally-curable nanocomposite printing for the scalable manufacturing of dielectric metasurfaces. Microsyst. Nanoeng..

[19] Choudhury SM (2018). Material platforms for optical metasurfaces. Nanophotonics.

[20] Wu Y, Yang W, Fan Y, Song Q, Xiao S (2019). TiO _2_ metasurfaces: from visible planar photonics to photochemistry. Sci. Adv..

[21] Langereis E, Roijmans R, Roozeboom F, van de Sanden MCM, Kessels WMM (2011). Remote plasma ALD of SrTiO_3_ using cyclopentadienlyl-based Ti and Sr precursors. J. Electrochem. Soc..

[22] Rose M (2009). Atomic layer deposition of titanium dioxide thin films from Cp*Ti(OMe)_3_ and ozone. J. Phys. Chem. C..

[23] Zydor A, Elliott SD (2011). TiCp*(OMe)_3_ versus Ti(OMe)_4_ in atomic layer deposition of TiO_2_ with water—ab initio modelling of atomic layer deposition surface reactions. J. Nanosci. Nanotech..

[24] Zydor A, Kessler VG, Elliott SD (2012). First principles simulation of reaction steps in the atomic layer deposition of titania: dependence of growth on Lewis acidity of titanocene precursor. Phys. Chem. Chem. Phys..

[25] Zydor A, Elliott SD (2010). Thermal stability of precursors for atomic layer deposition of TiO_2_, ZrO_2_, and HfO_2_: an ab initio study of α-hydrogen abstraction in bis-cyclopentadienyl dimethyl complexes. J. Phys. Chem. A.

[26] Niinistö J (2008). Novel mixed alkylamido-cyclopentadienyl precursors for ALD of ZrO_2_ thin films. J. Mater. Chem..

[27] Miyauchi M, Nakajima A, Watanabe T, Hashimoto K (2002). Photocatalysis and photoinduced hydrophilicity of various metal oxide thin films. Chem. Mater..

[28] Fujishima A, Zhang X, Tryk DA (2008). TiO_2_ photocatalysis and related surface phenomena. Surf. Sci. Rep..

[29] Levrau E (2013). Atomic layer deposition of TiO_2_ on surface modified nanoporous low-k films. Langmuir.

[30] Haider A, Yilmaz M, Deminskyi P, Eren H, Biyikli N (2016). Nanoscale selective area atomic layer deposition of TiO _2_ using e-beam patterned polymers. RSC Adv..

[31] Nguyen CT (2021). Atomic layer modulation of multicomponent thin films through combination of experimental and theoretical approaches. Chem. Mater..

[32] Gladfelter WL (1993). Selective metalization by chemical vapor deposition. Chem. Mater..

[33] Puurunen RL, Vandervorst W (2004). Island growth as a growth mode in atomic layer deposition: a phenomenological model. J. Appl. Phys..

[34] Guo L, Oskam G, Radisic A, Hoffmann PM, Searson PC (2011). Island growth in electrodeposition. J. Phys. D: Appl. Phys..

[35] Parsons GN (2019). Functional model for analysis of ALD nucleation and quantification of area-selective deposition. J. Vac. Sci. Technol. A.

[36] Avrami M (1940). Kinetics of phase change. II Transformation‐time relations for random distribution of nuclei. J. Chem. Phys..

[37] Ko, B. G. et al. Growth modulation of atomic layer deposition of HfO_2_ by combinations of H_2_O and O_3_ reactants. *Dalton Trans*. 10.1039/D1DT03465K (2021). 10.1039/d1dt03465k 34821888

[38] Cho Y, Kim SH, Kim BS, Kim Y, Jeon W (2021). Modulation of the adsorption chemistry of a precursor in atomic layer deposition to enhance the growth per cycle of a TiO_2_ thin film. Phys. Chem. Chem. Phys..

[39] Gordon RG, Hausmann D, Kim E, Shepard J (2003). A kinetic model for step coverage by atomic layer deposition in narrow holes or trenches. Chem. Vap. Depos..

[40] Nguyen CT, Yoon J, Khan R, Shong B, Lee H-B-R (2019). Thermal atomic layer deposition of metallic Ru using H_2_O as a reactant. Appl. Surf. Sci..

[41] Heikkilä M, Puukilainen E, Ritala M, Leskelä M (2009). Effect of thickness of ALD grown TiO_2_ films on photoelectrocatalysis. J. Photochemistry Photobiol. A: Chem..

[42] Xie Q (2008). Growth kinetics and crystallization behavior of TiO_2_ films prepared by plasma enhanced atomic layer deposition. J. Electrochem. Soc..

[43] Gonzalez-Ramirez JD, Villicañamendez M, Tiznado H, Alonso-Nuñez G, Cortes JA (2018). Atomic layer deposition of TiO_2_ from tetrakis (dimethylamino) titanium and H_2_O on commercial-grade iron: a simple method for support preparation. Int. Referee. J. Eng. Sci..

[44] Zhuiykov S (2017). Wafer-scale fabrication of conformal atomic-layered TiO_2_ by atomic layer deposition using tetrakis (dimethylamino) titanium and H_2_O precursors. Mater. Des..

[45] Reinke M, Kuzminykh Y, Hoffmann P (2015). Surface kinetics of titanium isopropoxide in high vacuum chemical vapor deposition. J. Phys. Chem. C..

[46] Filipovic, L. Modeling and simulation of atomic layer deposition. In *2019 International Conference on Simulation of Semiconductor Processes and Devices (SISPAD)* 1–4 (IEEE, 2019).

[47] Kresse G, Furthmüller J (1996). Efficient iterative schemes for ab initio total-energy calculations using a plane-wave basis set. Phys. Rev. B.

[48] Perdew JP, Burke K, Ernzerhof M (1996). Generalized gradient approximation made simple. Phys. Rev. Lett..

[49] Grimme S, Ehrlich S, Goerigk L (2011). Effect of the damping function in dispersion corrected density functional theory. J. Computational Chem..

[50] Dudarev SL, Botton GA, Savrasov SY, Humphreys CJ, Sutton AP (1998). Electron-energy-loss spectra and the structural stability of nickel oxide: an LSDA+U study. Phys. Rev. B.

[51] Hu Z, Metiu H (2011). Choice of *U* for DFT+ *U* calculations for titanium oxides. J. Phys. Chem. C..

[52] Cheng H-E, Chen C-C (2008). Morphological and photoelectrochemical properties of ALD TiO_2_ films. J. Electrochem. Soc..

[53] Lazzeri M, Vittadini A, Selloni A (2001). Structure and energetics of stoichiometric TiO_2_ anatase surfaces. Phys. Rev. B.

[54] Walle LE (2011). Mixed dissociative and molecular water adsorption on anatase TiO_2_ (101). J. Phys. Chem. C..

[55] Henkelman G, Uberuaga BP, Jónsson H (2000). A climbing image nudged elastic band method for finding saddle points and minimum energy paths. J. Chem. Phys..

[56] Henkelman G, Jónsson H (2000). Improved tangent estimate in the nudged elastic band method for finding minimum energy paths and saddle points. J. Chem. Phys..

