# Publisher Correction: Gradient area-selective deposition for seamless gap-filling in 3D nanostructures through surface chemical reactivity control

**DOI:** 10.1038/s41467-023-35883-9

**Published:** 2023-01-12

**Authors:** Chi Thang Nguyen, Eun-Hyoung Cho, Bonwook Gu, Sunghee Lee, Hae-Sung Kim, Jeongwoo Park, Neung-Kyung Yu, Sangwoo Shin, Bonggeun Shong, Jeong Yub Lee, Han-Bo-Ram Lee

**Affiliations:** 1 Department of Materials Science and Engineering, Incheon National University, Incheon, 22012 Korea; 2 Beyond Silicon Lab, Samsung Advanced Institute of Technology, Gyeonggi, 16678 Korea; 3 Department of Chemical Engineering, Hongik University, Seoul, 04066 Korea; 4 Department of Mechanical and Aerospace Engineering, University at Buffalo, Buffalo, NY 14260 USA

**Keywords:** Synthesis and processing, Chemical engineering

Correction to: *Nature Communications* 10.1038/s41467-022-35428-6, published online 09 December 2022

The original version of this Article incorrectly made the peer review reports for this paper available. In the correct HTML version, the link to the peer review file has been removed. The second sentence of the Peer Review Information section, which stated ‘Peer reviewer reports are available’ has been removed. This has been corrected in both the PDF and HTML versions of the Article.

