# Supplementary material for: Gradient area-selective deposition for seamless gap-filling in 3D nanostructures through surface chemical reactivity control
Source: Nat Commun. 2022 Dec 9;13:7597. doi: 10.1038/s41467-022-35428-6 (PMC9734176; doi:10.1038/s41467-022-35428-6)
Supplement: Supplementary file 1 — Supplementary Information [file 41467_2022_35428_MOESM1_ESM.pdf]

Supplementary Information

Gradient Area-Selective Deposition for Seamless Gap-filling in 3D Nanostructures through Surface Chemical Reactivity Control

Nguyen et al.

Supplementary Information

Supplementary Note 1: Dissociative adsorption mechanism of TMPMCT on TiO_2 surface.

The adsorption energy of TMPMCT on the TiO_2 surface is $E_{ad} = -1.52$ eV and the dissociation of the OMe ligands can proceed via multiple reaction paths. Among possible routes, the most probable two paths were considered in the study, Path 1 and Path 2 (sub–paths 2-1 and 2-2). In the Path 1, the dissociation of first OMe ligand takes place (activation energy $E_a = 0.46$ eV, energy $E = -2.02$ eV), followed by the second OMe dissociation ($E_a = 0.70$ eV, $E = -2.23$ eV). Similarly, Path 2 results in the first OMe dissociation but requires higher energy than Path 1 ($E_a = 0.82$ eV, $E = -1.90$ eV), from which the path diverges to sub–paths 2-1 and 2-2. Path 2-1 leads to the surface diffusion of adsorbed TMPMCT to the adjacent site first to be more stabilised ($E_a = 0.07$ eV, $E = -3.15$ eV) and then the dissociation of the second OMe ligand ($E = -2.04$ eV). In the more endothermic Path 2-2, TMPMCT diffuses and loses the second OMe ligand concomitantly ($E = -1.26$ eV).

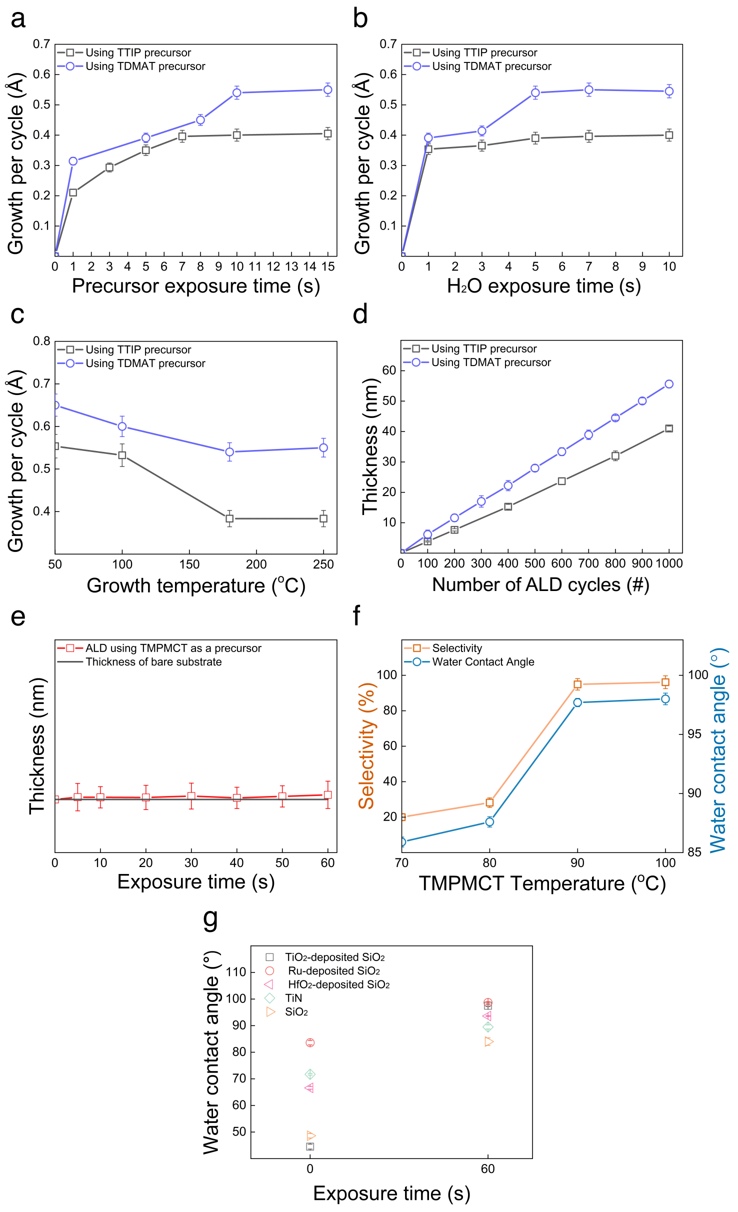

Supplementary Fig. 1: Growth characteristics of TiO_2 ALD films using TTIP and TDMAT precursor with an H_2O counter-reactant. **a**, Effect of precursor exposure time on film GPC. **b**, Effect of H_2O reactant exposure time. **c**, ALD window and **d**, thickness as a function of ALD cycle. **e**, Thickness of ALD-grown TiO_2 thin film using TMPMCT as the precursor and H_2O as the reactant. **f**, Selectivity and WCA at various TMPMCT temperatures. **g,** Adsorption behaviour of TMPMCT when exposed on five substrates, namely, TiO_2-deposited SiO_2, Ru-deposited SiO_2,

HfO_2-deposited SiO_2, TiN, and SiO_2. The TMPMCT inhibitor was exposed for 60 s on these substrates; the WCA increased. Source data are provided as a Source Data file.

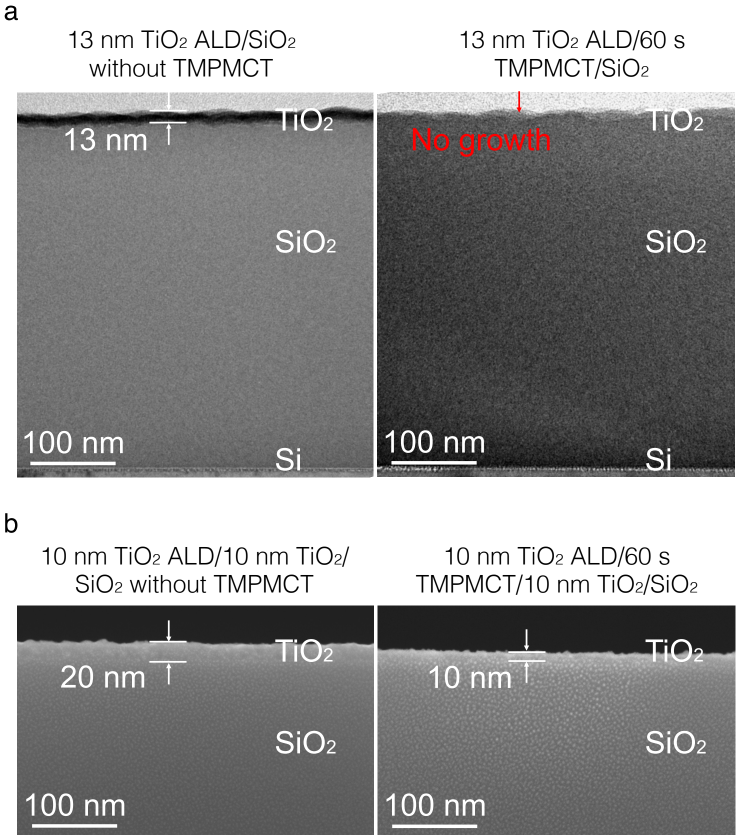

Supplementary Fig. 2: a, Blocking property of TMPMCT against TiO_2 ALD on the SiO_2 substrate; 13 nm TiO_2 growth occurs on the bare SiO_2 surface, but not on the TMPMCT-inhibited SiO_2 surface. **b,** In the case of 10 nm TiO_2-deposited SiO_2 substrate, there is also no growth of subsequent TiO_2 ALD on TMPMCT-inhibited TiO_2/SiO_2 surface, meanwhile, the total thickness of TiO_2 is 20 nm after 10 nm TiO_2 ALD on bare 10 nm TiO_2/SiO_2.

Supplementary Table 1: Highest inhibition of the TiO_2 ALD film growth (TDMAT precursor) herein and that in the literature.

TiO_2 Precursor	Reactant	Deposition Temp. (°C)	Blocking layer	Thickness of the thickest inhibited TiO_2 film (nm)	Reference
TDMAT	H_2O	150	43 nm *poly(methylmethacrylate) (PMMA)*	50	[1]
TDMAT	H_2O	150	76 nm polynorbornene (PNB)	48	[2]
TDMAT	H_2O	180	Single layer of TMPMCT inhibitor	45	This study

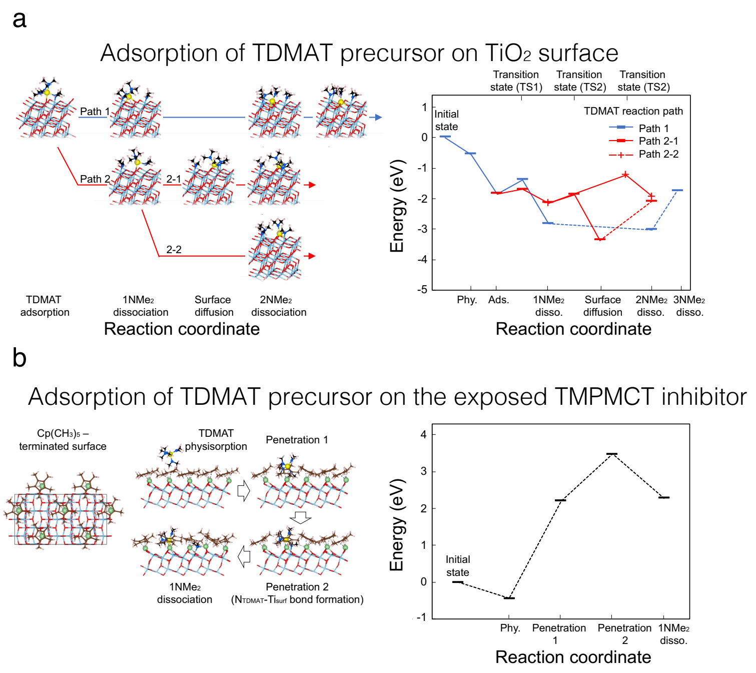

Supplementary Fig. 3: **a**, Reactivity of the TiO_2 precursor (TDMAT) on the TiO_2 surface. The corresponding structures (left) and energy diagram (right), calculated by DFT. The dissociation/binding reaction of the NMe_2 ligand, which has the most stable energy, is considered the main reaction pathway. Among the considered routes, only the most favourable two paths were plotted, Path 1 and Path 2 (sub–paths 2–1 and 2–2). After physisorption on the TiO_2 surface (E = −0.55 eV), TDMAT adsorbs to the surface, where Ti_{prec}-O_{surf} and N_{prec}-Ti_{surf} bonds are formed without ligand dissociation (E = −1.84 eV). Afterwards, the dissociation of the first NMe_2 ligand take place (Path 1: E_a = 0.46 eV, E = −2.82 eV; Path 2: E_a = 0.16 eV, E = −2.14 eV). In the Path 1, the energy after the dissociation of the second and third NMe_2 ligands are −3.02 and −1.73 eV. The Path 2 diverges to two sub–paths, where in the Path 2-1, the adsorbed TDMAT diffuses to an adjacent adsorption site first to be more stabilized (E_a = 0.27 eV, E = −3.37 eV) and then loses the second NMe_2 ligand (E = −2.09 eV), or in the Path 2-2, the adsorbed TDMAT diffuses and loses the second NMe_2 ligand concomitantly (E_a = 0.93 eV, E = −1.92 eV). **b**, Adsorption of TDMAT on the $Cp(CH_3)_5$–terminated surface, calculated by DFT. The structure of the $Cp(CH_3)_5$-terminated

surface and the corresponding surface structures (left), energy diagram (right). After physisorption (E = −0.44 eV), TDMAT penetrates into the $Cp(CH_3)_5$-dense overlayer (E = 2.20 eV). Afterward, TDMAT enters deeper to form N_{TDMAT}-Ti_{surf} bond without NMe_2 dissociation (3.47 eV). As the reaction proceeds further, a NMe_2 ligand dissociates from TDMAT to form Ti_{TDMAT}−O_{surf} bond (E = 2.28 eV). The chemisorption of TDMAT on the $Cp(CH_3)_5$−terminated surface requires a significantly higher energy than the clean surface. The dotted lines in the energy diagrams represent the reactions, where transition states (TS) structures were not calculated. Only Ti_{TMPMCT}, Ti_{TDMAT}, O_{TMPMCT}, N_{TDMAT} atoms are depicted with ball model. Colours: Ti_{surf} (light blue), Ti_{TMPMCT} (cyan), Ti_{TDMAT} (pink), O (red), N (blue), C (brown), H (light pink). Abbreviations: prec (precursor), surf (surface).

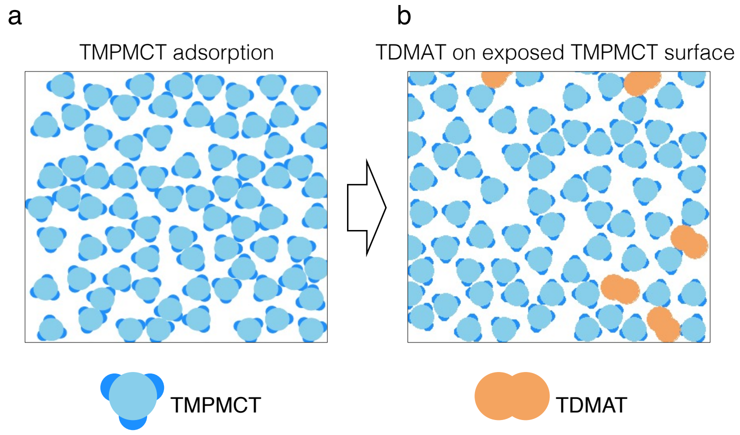

Supplementary Fig. 4: a, The saturated surface of TMPMCT still has unoccupied sites due to the steric interference. **b,** The unoccupied sites on exposed TMPMCT surface are potential sites for the next TDMAT precursor adsorption.

Supplementary Note 2: The kinetic model based on the Johnson−Mehl−Avrami−Kolmogorov (JMAK) model.

The model is composed of several equations: (1) growth initiation and film nucleation, (2) fraction of the growth surface covered with a film versus ALD cycles, (3) approximate calculated film thickness in the non-growth area, and (4) selectivity. Details for the JMAK model can be found in[3–5].

$$\dot{N}(n) = \dot{N}_0 \exp(-v_d/n) \quad (1)$$

$$A_f/A_0 = 1 - \exp(-A_e/A_0) \quad (2)$$

In which, $A_e(n) = A_0\pi\left(\dot{G}n\right)^2\widehat{N} + A_0\pi\int_0^n[\dot{G}(n-v)]^2\,\dot{N}_0\exp(-v_d/v)\,\partial v \quad (3)$

$$\tilde{t}_{ng}(n) = \frac{1}{A_0}\int_0^n\left(2-\frac{A_f}{A_0}\right)A_f\dot{G}\,dn \quad (4)$$

$$S(n) = \left(1-\frac{A_f}{A_0}\right)/\left(1+\frac{A_f}{A_0}\right) \quad (5)$$

where, $\dot{N}(n)$ is the nucleation site generation rate for the non-growth area after n cycles (nm^{-2} cycle^{-1}), $\dot{N}_0$ is the nucleation site generation rate for the non-growth area (nm^{-2} cycle^{-1}), v_d is the nucleation site generation delay cycles for the non-growth area (cycles), *n* is the number of ALD cycles (cycle), A_f is the area of the substrate covered by the film (nm^2), A_0 is the area of the substrate (nm^2), A_e is the extended area (nm^2), $\dot{G}$ is the growth rate for a material depositing on itself (nm cycle^{-1}), $\widehat{N}$ is the nucleation site density on a starting non-growth area (nm^{-2}), *v* is the cycles when nucleus starts to grow (cycle), $\tilde{t}_{ng}$ is the approximate calculated film thickness on the non-growth area (nm), and *S* is the selectivity (%). $\dot{G}$ was obtained from experiments as 0.055 nm cycle^{-1}, as shown in Fig. 2c and Supplementary Fig. 1a–1d.

In this study, we considered cycle number n ranging from 0 to 1000, based on the experiments. Nucleation site density was defined based on the following assumptions: there is no growth site in the starting non-growth area and nucleation site density $\widehat{N} = 0$ when $n = 0$. No nuclei formation was observed in the first cycles. The calculations were repeated in cycle increments (1

cycle/step) until the selectivity dropped below 90% at a v_d cycle, which was recorded as a delay cycle. From this delay cycle (v_d) onwards, the nuclei were assumed to be randomly distributed with fixed positions on the surface, which were not covered by the inhibitor. In other words, nucleation sites were generated as a function of cycle number and nuclei site generation rate ($\dot{N}_0$); the relation is shown in equation (1).

The nuclei size evolution was uniform in all directions with constant growth per cycle ($\dot{G}$), which was determined from the experimental results as 0.055 nm/cycle. After n cycles, each growth site would have grown into a circle with radius of $\dot{G} \times n$. The extended area, A_e, due to the nuclei formation sites is the product of area of each nucleus and number of nuclei present, calculated using equation (3). As a result, the coverage of the initial substrate surface (A_f/A_0) by the growing film varies with ALD growth cycles and is a function of $\dot{N}_0$, v_d, and $\dot{G}$ (schematic in Supplementary Fig. 5). The coverage was calculated using equation (2).

The coverage of TMPMCT varied with the TMPMCT inhibitor exposure time, resulting in different nuclei site generation delay cycles (v_d) and $\dot{N}_0$. For instance, because the 20 s TMPMCT sample had the lowest coverage, it had a lower v_d and a higher $\dot{N}_0$ than the 40 and 60 s samples. After evaluating parameters $\dot{N}_0$, v_d, $\dot{G}$, the approximate film thickness, $\tilde{t}_{ng}(n)$, on the non-growth area and the selectivity, $S(n)$, for each ALD cycle can be estimated using equation (4) and equation (5), respectively.

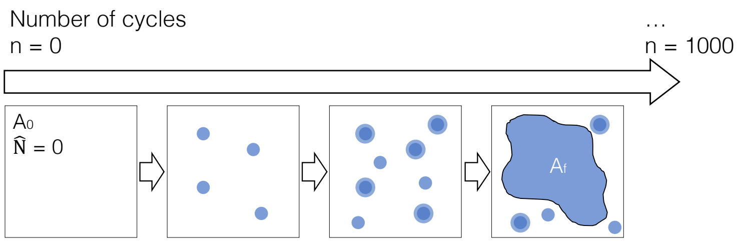

Supplementary Fig. 5: Schematic of continuous nuclei generation on the TMPMCT inhibition layer/TiO_2 surface with increasing number of TiO_2 ALD cycles.

Supplementary Table 2: Parameters for the JMAK model shown in Fig. 2 and Supplementary Fig. 6.

Case	Parameters			
	$\dot{G}$ (nm·cycle^{-1})	$\widehat{N}$ (nm^{-2})	$\dot{N}_0$ (nm^{-2}·cycle^{-1})	ν_d (cycles)
20 s TMPMCT	0.055	0	68.0×10^{-7}	80
40 s TMPMCT	0.055	0	4.9×10^{-7}	620
60 s TMPMCT	0.055	0	2.2×10^{-7}	800

The extracted parameters for the nucleation site generation rate, $\dot{N}_0$, were 68.0×10^{-7}, 4.9×10^{-7}, and 2.2×10^{-7} nm^{-2} cycle^{-1} for 20, 40, and 60 s samples, respectively. $\dot{N}_0$ of the 20 s sample was 31 times and 14 times larger than that of the 60 s sample and 40 s sample, respectively.

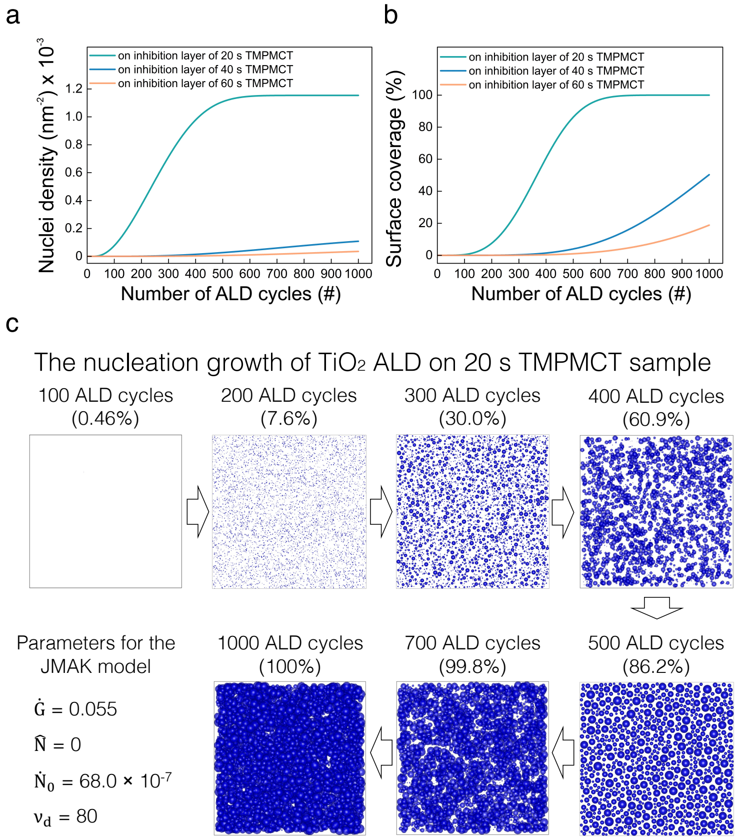

Supplementary Fig. 6: a, Nuclei density and **b**, surface coverage vs ALD cycles with different TMPMCT exposure times (20, 40, and 60 s) as simulated by the JMAK Model. **c**, Nucleation growth of TiO_2 on the 20 s TMPMCT inhibitor layer simulated by the JMAK model. Source data are provided as a Source Data file.

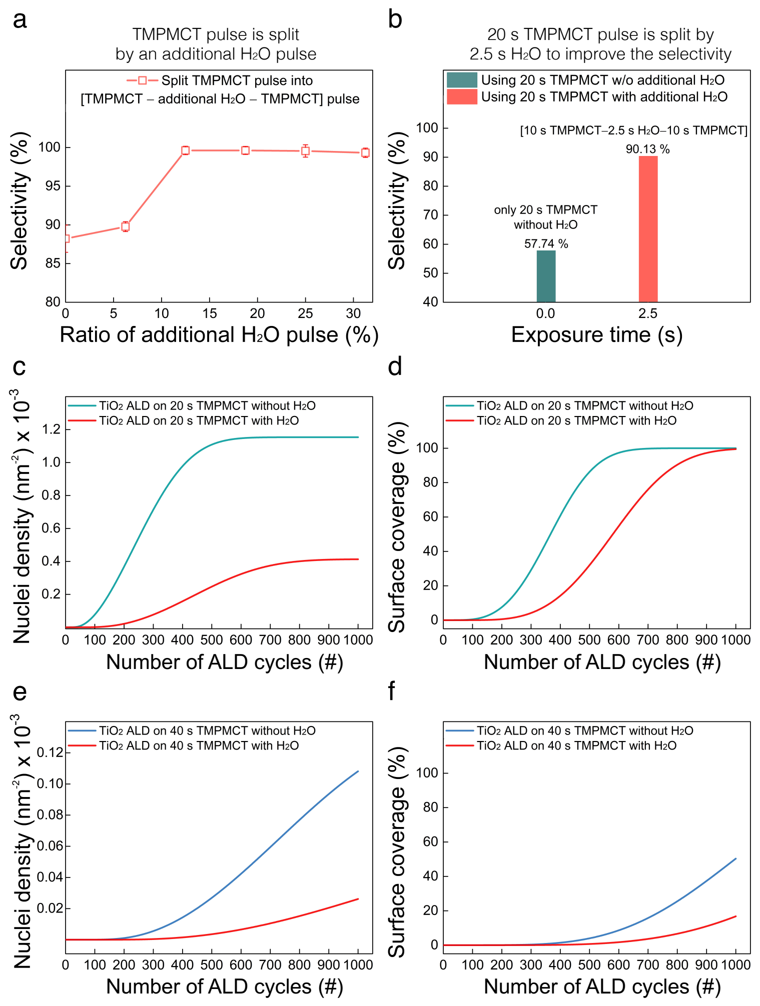

Supplementary Fig. 7: TMPMCT pulse modified by adding an additional H_2O pulse. **a**, Optimal time of additional H_2O over the entire TMPMCT pulse; the 40 s TMPMCT inhibitor sample is modified into a [20 s TMPMCT – additional H_2O – 20 s TMPMCT] pulse to inhibit the growth during 800 ALD cycles; selectivity of the 40 s TMPMCT inhibitor sample is 88.2%, and improves to 99.6% with an additional 5 s H_2O pulse, i.e., 12.5% compared to 40 s of the entire TMPMCT pulse. **b**, Optimisation applied to the 20 s TMPMCT pulse; the 20 s TMPMCT pulse modified into [10 s TMPMCT – 2.5 s additional H_2O – 10 s TMPMCT] pulse to inhibit the growth during 400 ALD cycles; selectivity is 90.1% in relation to 57.7% in the case of only the 20 s TMPMCT inhibitor sample. **c**, Nuclei density and **d**, surface coverage as a function of ALD cycles in the case

of [10 s TMPMCT – 2.5 s H_2O – 10 s TMPMCT] pulse. **e**, Nuclei density and **f**, Surface coverage as a function of ALD cycles in the case of [20 s TMPMCT –5 s H_2O – 20 s TMPMCT] pulse. Source data are provided as a Source Data file.

Supplementary Table 3: Parameters for the JMAK model shown in Fig. 3.

	Parameters			
Case	$\dot{G}$ (nm·cycle^{-1})	$\hat{N}$ (nm^{-2})	$\dot{N}_0$ (nm^{-2}·cycle^{-1})	ν_d (cycle)
[10 s TMPMCT – 2.5 s H_2O – 10 s TMPMCT]	0.055	0	2.8×10^{-7}	400
[20 s TMPMCT – 5 s H_2O – 20 s TMPMCT]	0.055	0	1.9×10^{-7}	900

The parameters obtained from the best fit results of the JMAK model, including $\dot{G}$, $\dot{N}_0$, and ν_d, are listed in Supplementary Table 2, and the fitting results with the experimental data are plotted in Fig. 3d–3g. The extracted parameters for the nucleation site generation rate of the TMPMCT with additional H_2O samples, $\dot{N}_0$, are 2.8×10^{-7} and 1.9×10^{-7} nm^{-2} cycle^{-1} for 20 s and 40 s, respectively. The nucleation delays from the fittings of TMPMCT with additional H_2O are 400 and 900 cycles for 20 s and 40 s, respectively.

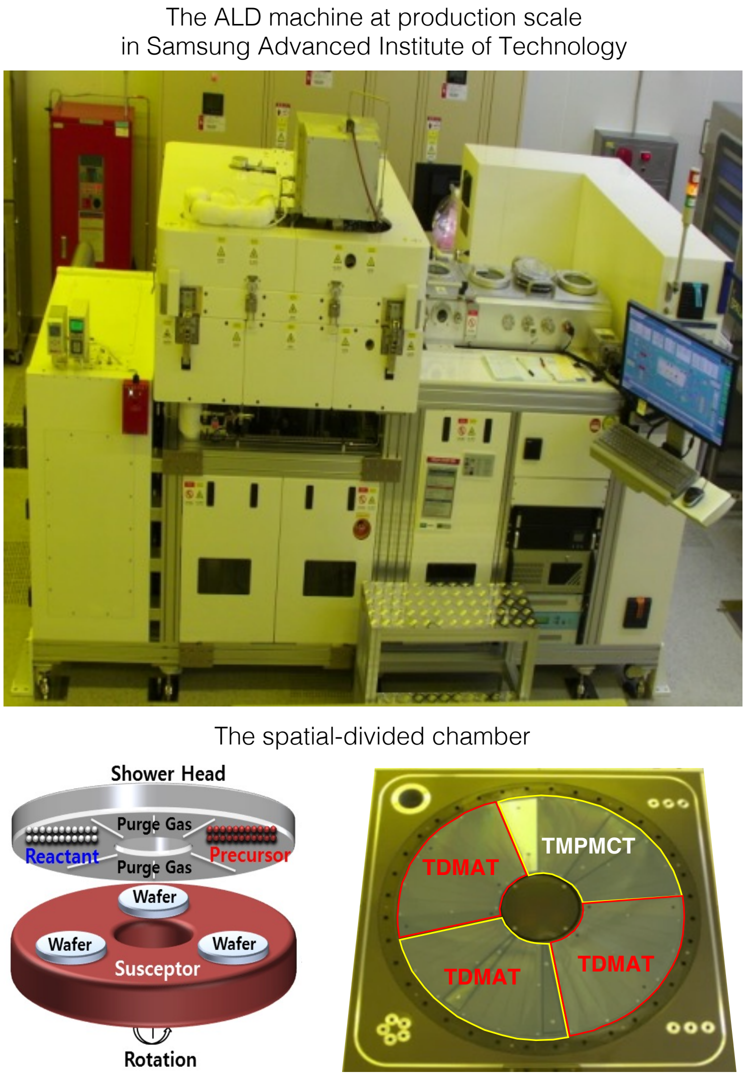

Supplementary Fig. 8: ALD machine at the production scale in the Samsung Advanced Institute of Technology.

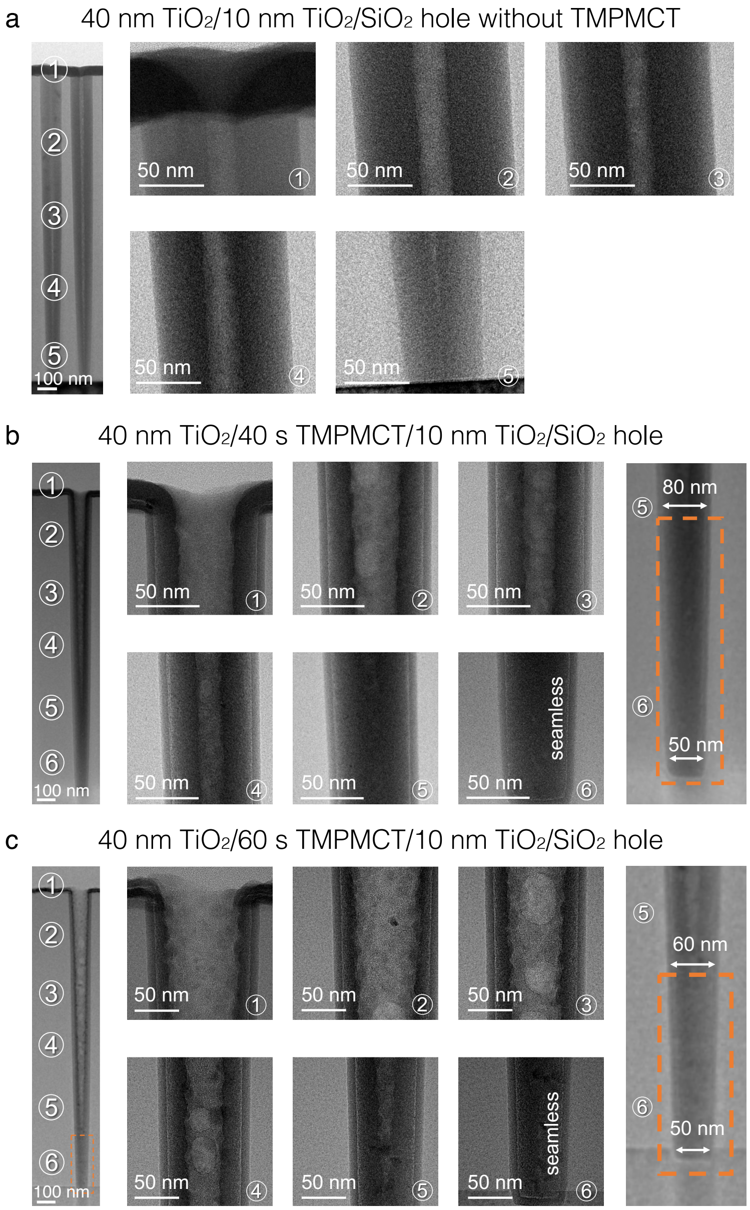

Supplementary Fig. 9: a, TEM images of a typical 40 nm TiO_2 ALD/10 nm TiO_2/SiO_2 hole without TMPMCT, revealing an unfilled zone and seam formation along the centreline of the hole. When the TMPMCT is exposed up to **b,** 40 s and **c,** 60 s on the 10 nm TiO_2/SiO_2 hole, it can inhibit the growth of 40 nm TiO_2 on the top of hole; the blocking property degrades with increasing hole depth.

Supplementary Note 3: Kinetic model used to estimate the adsorption density of TMPMCT inside 3D nanoscale holes.

Based on the simple case (planar substrate) mentioned in the kinetic description about the number of molecules crossing a plane from one side per unit time and unit area in an ALD, we suggest the time t (s), which is required to cover the surface area of a 3D hole down to the depth λ (nm). In an ideal ALD process, we assume that the sticking coefficient of TMPMCT on TiO_2 surface is 100%, the time t (s) can be calculated by the following equation[6]:

$$t = \int_0^t \mathrm{d}t = \frac{Q\sqrt{2\pi mkT}}{P}\frac{p}{A_{hole}}\int_0^L \mathrm{d}\lambda\left(1 + \frac{3p\lambda}{16\mathrm{A}_{\mathrm{hole}}}\right)$$

where P is the partial pressure of the precursor near the surface (Pa), Q is the number of molecules (saturation dose) that can be adsorbed on a square metre, m is the molecular mass (kg), k is the Boltzmann constant (1.38×10^{-23} J/K), T is the temperature (K), p is the perimeter of the hole (nm), L is the depth of the hole (nm), and $\mathrm{A}_{\mathrm{hole}}$ is the cross-sectional area (nm^2).

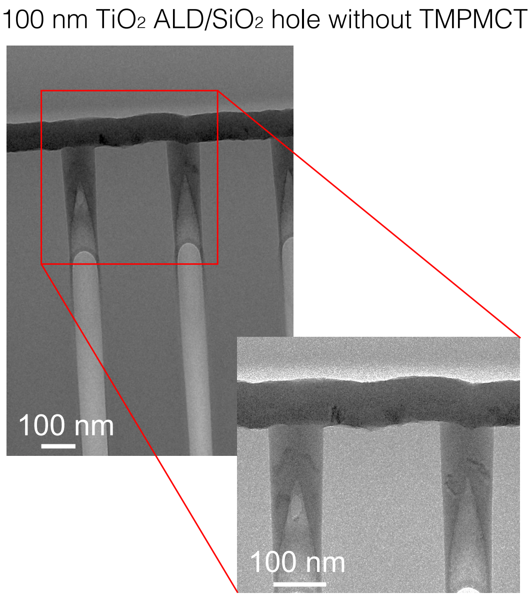

Supplementary Fig. 10: TEM image of a 100 nm TiO_2 ALD/SiO_2 hole without TMPMCT, revealing larger voids.

a 100 nm TiO_2 gap-filling using 60 s TMPMCT

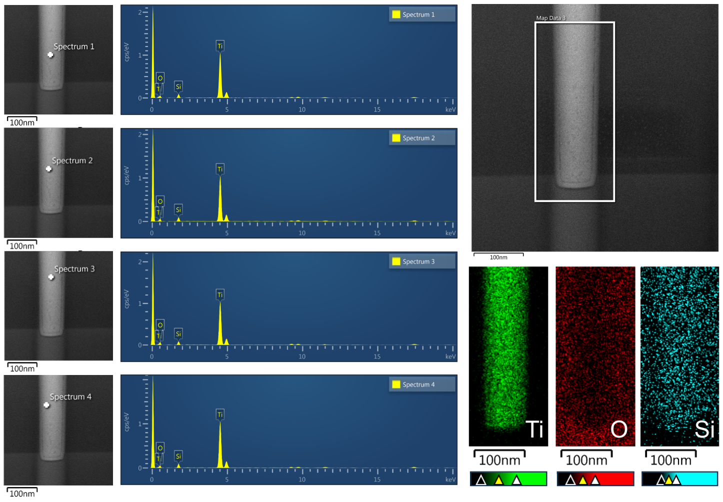

b Component ratios from EDS mapping

	Positions			
	S1	S2	S3	S4
O	8.83	11.95	12.10	8.46
Si	7.52	7.76	7.14	8.12
Ti	83.65	80.29	80.76	83.42

Supplementary Fig. 11: The high-aspect-ratio hole (depth = 1600 nm) was filled by TiO_2 gradient ASD using 60 s TMPMCT. **a,** TEM images with EDS mapping results obtained at different positions show that TiO_2 fills the gaps without seam formation. **b,** Component ratios obtained from EDS mapping.

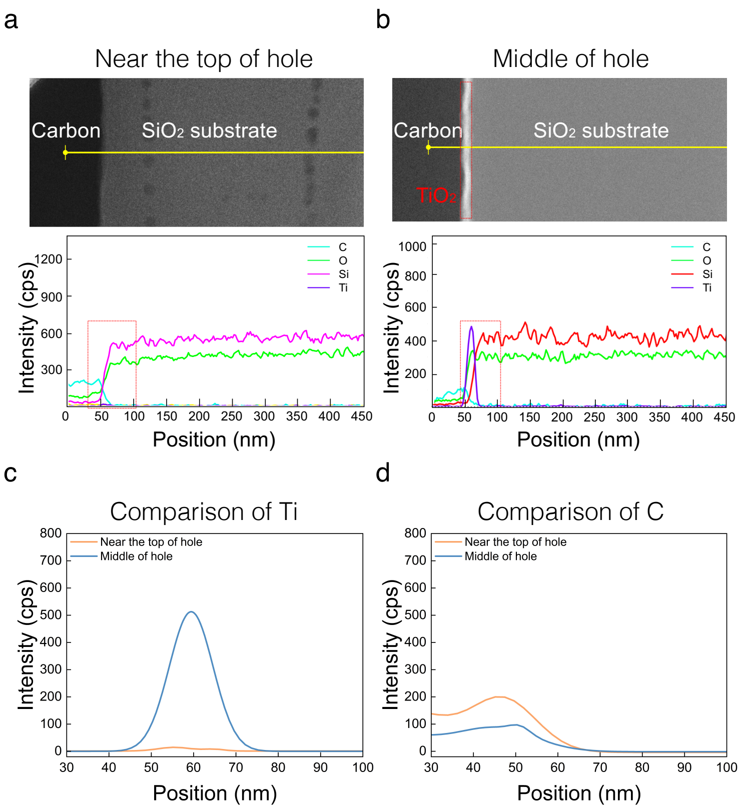

Supplementary Fig. 12: TEM image with EDS line profile across the ASD TiO_2/TMPMCT/SiO_2 sample. **a,** Near the top of the hole, there is no growth of TiO_2 due to the high density of adsorbed TMPMCT. **b**, In the middle of the hole, the growth of TiO_2 thin film is observed. As a result, the intensity of the Ti peak is increased. **c**, Comparison results reveal a small amount of Ti in the top region, attributed to either the presence of the TMPMCT layer or early TiO_2 nucleation growth. In the 50–70 nm position, Ti peak intensity is high in the middle region, because of the TiO_2 thin film formation. **d,** Comparison of C intensities. Owing to the existence of the C protection layer for TEM analysis, C signals are detected for both regions. At the middle region of the hole, including the TiO_2 growth layer, the decrease in the C peak intensity implies the presence of negligible Ti–C impurities.

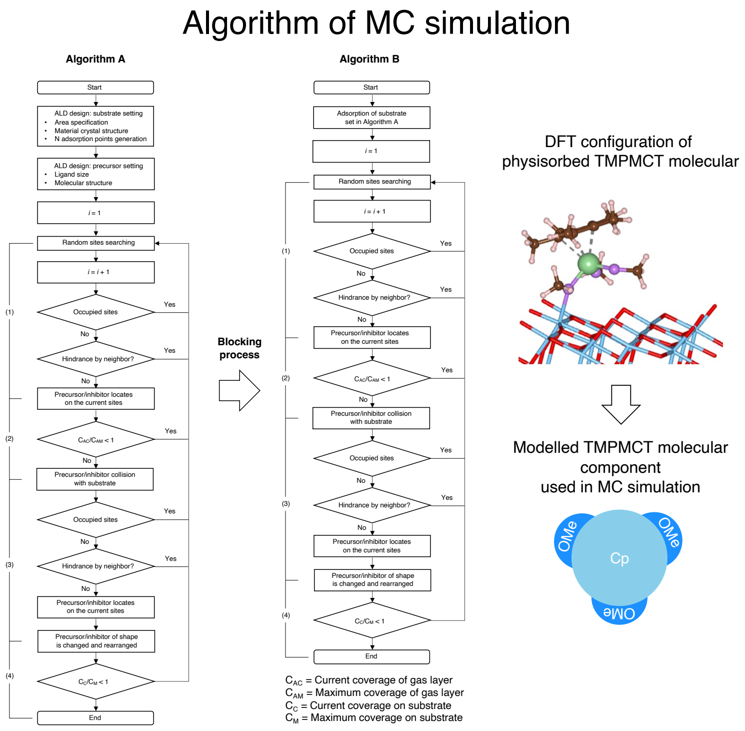

Supplementary Fig. 13: Algorithm of Monte Carlo simulation[7]. The sizes of molecular components were modelled using the MOLview tool[8–10], assuming a spherical model. The TMPMCT molecular is assumed to occupy only one potential site; the point and areal coverages of precursor molecules are described and calculated according to the following formulae:

$$\text{Point coverage (\%)} = \frac{\text{total number of molecular adsorptions}}{\text{total number of adsorption sites}}$$

$$\text{Areal coverage (\%)} = \frac{\text{total number of molecular adsorptions} \times \text{molecular size}}{\text{substrate size}}$$

Supplementary Table 4: Raw data for calculating the thickness and selectivity in the cases of 20 s, 40 s, and 60 s TMPMCT exposures and 40 s TMPMCT exposure with an additional H_2O pulse.

Typical TiO_2 ALD

	ALD cycle	Thickness (Å)/ Run number									
		1	2	3	4	5	6	7	avg	std	unif(σ)
Typical TiO_2 ALD	100	60.8	58.8	59.5	61.7	60.9	60.8	61.7	60.6	1.07	1.8%
	200	115.2	110.1	113.0	117.1	116.4	115.1	115.8	114.7	2.39	2.1%
	300	169.2	162.1	165.7	170.9	170.1	169.3	169.5	168.1	3.13	1.9%
	400	221.3	211.3	216.2	223.6	221.9	221.0	221.0	219.5	4.26	1.9%
	500	278.8	266.1	272.4	282.3	279.9	278.4	277.6	276.5	5.50	2.0%
	600	333.1	319.6	326.4	335.9	333.9	332.3	330.8	330.3	5.57	1.7%
	700	388.6	372.8	380.8	391.9	389.5	387.7	385.9	385.3	6.50	1.7%
	800	444.1	426.1	435.2	447.9	445.2	443.1	441.0	440.4	7.42	1.7%
	900	499.7	479.4	489.6	503.8	500.8	498.5	496.1	495.4	8.35	1.7%
	1000	555.2	532.6	544.1	559.8	556.5	553.9	551.3	550.5	9.28	1.7%

Selectivity in the case of [20 s TMPMCT]

	ALD cycle	Thickness (Å)/ Run number									
		1	2	3	4	5	6	7	avg	std	unif(σ)
ALD on [20 s TMPMCT]	100	10.4	8.5	10.1	11.0	10.7	10.2	10.7	10.2	0.82	8.1%
	200	15.7	15.1	16.0	15.4	15.6	15.9	16.1	15.7	0.35	2.2%
	300	13.6	12.7	14.2	13.8	13.7	14.1	14.7	13.8	0.63	4.6%
	400	12.4	10.8	12.7	12.6	12.5	13.0	13.8	12.5	0.90	7.2%
	500	42.2	3.5	2.7	21.6	33.4	44.6	46.5	27.8	18.83	67.8%
	600	113.6	58.7	11.2	92.5	105.3	111.4	111.7	86.3	38.35	44.4%
	700	167.6	109.0	11.0	148.4	159.2	168.1	169.5	133.3	57.94	43.5%
	800	227.1	170.4	45.4	207.8	218.5	228.5	229.4	189.6	66.88	35.3%
	900	299.5	241.5	128.4	283.6	292.9	299.0	304.7	264.2	63.56	24.1%
	1000	369.3	311.2	203.8	351.5	362.4	368.0	369.8	333.7	60.91	18.3%
Selectivity [20 s TMPMCT]	100	0.71	0.75	0.71	0.70	0.70	0.71	0.70	0.71	0.017	2.3%
	200	0.76	0.76	0.75	0.77	0.76	0.76	0.76	0.76	0.005	0.7%
	300	0.85	0.85	0.84	0.85	0.85	0.85	0.84	0.85	0.005	0.6%
	400	0.89	0.90	0.89	0.89	0.89	0.89	0.88	0.89	0.006	0.7%
	500	0.74	0.97	0.98	0.86	0.79	0.72	0.71	0.82	0.115	13.9%
	600	0.49	0.69	0.93	0.57	0.52	0.50	0.50	0.60	0.163	27.2%
	700	0.40	0.55	0.94	0.45	0.42	0.40	0.39	0.51	0.201	39.6%
	800	0.32	0.43	0.81	0.37	0.34	0.32	0.32	0.42	0.179	43.1%
	900	0.25	0.33	0.58	0.28	0.26	0.25	0.24	0.31	0.123	39.3%
	1000	0.20	0.26	0.45	0.23	0.21	0.20	0.20	0.25	0.093	37.0%

Selectivity in the case of [40 s TMPMCT]

	ALD cycle	Thickness (Å)/ Run number									
		1	2	3	4	5	6	7	avg	std	unif(1σ)
ALD on [40 s TMPMCT]	100	3.3	2.8	3.2	3.3	3.6	4.1	4.3	3.5	0.51	14.6%
	200	9.3	10.2	3.3	1.6	7.8	9.8	8.5	7.2	3.39	47.1%
	300	8.1	8.7	7.9	7.3	7.3	8.7	8.6	8.1	0.62	7.6%
	400	7.5	7.9	7.4	7.0	6.9	8.1	7.6	7.5	0.43	5.7%
	500	5.6	5.7	5.3	4.8	4.7	6.0	5.6	5.4	0.46	8.5%
	600	0.9	1.7	1.7	1.1	1.1	0.9	0.8	1.2	0.39	32.8%
	700	0.9	1.1	1.9	1.7	2.1	0.6	0.5	1.3	0.63	49.6%
	800	4.3	2.5	3.5	3.2	1.5	8.7	16.8	5.8	5.36	93.1%
	900	47.3	8.6	11.2	0.9	31.3	57.8	72.7	32.8	27.39	83.4%
	1000	149.3	46.9	11.8	90.1	132.2	154.4	164.2	107.0	59.01	55.2%
Selectivity [40 s TMPMCT]	100	0.90	0.91	0.90	0.90	0.89	0.87	0.87	0.89	0.014	1.6%
	200	0.85	0.83	0.94	0.97	0.87	0.84	0.86	0.88	0.054	6.1%
	300	0.91	0.90	0.91	0.92	0.92	0.90	0.90	0.91	0.008	0.8%
	400	0.93	0.93	0.93	0.94	0.94	0.93	0.93	0.93	0.004	0.5%
	500	0.96	0.96	0.96	0.97	0.97	0.96	0.96	0.96	0.004	0.4%
	600	0.99	0.99	0.99	0.99	0.99	0.99	0.99	0.99	0.002	0.2%
	700	1.00	0.99	0.99	0.99	0.99	1.00	1.00	0.99	0.003	0.3%
	800	0.98	0.99	0.98	0.99	0.99	0.96	0.93	0.97	0.023	2.4%
	900	0.83	0.96	0.96	1.00	0.88	0.79	0.74	0.88	0.096	10.9%
	1000	0.58	0.84	0.96	0.72	0.62	0.56	0.54	0.69	0.159	23.1%

Selectivity in the case of [60 s TMPMCT]

	ALD cycle	Thickness (Å)/ Run number									
		1	2	3	4	5	6	7	avg	std	unif(1σ)
ALD on [60 s TMPMCT]	100	4.1	4.3	3.6	3.1	3.6	4.2	4.1	3.9	0.45	11.7%
	200	10.1	11.0	10.4	8.6	9.7	11.0	11.4	10.3	0.96	9.3%
	300	9.4	11.4	10.3	9.5	9.9	9.8	9.7	10.0	0.67	6.7%
	400	9.2	11.1	9.9	9.1	9.8	9.8	9.7	9.8	0.65	6.7%
	500	7.3	8.9	7.4	6.0	6.9	7.2	7.1	7.3	0.86	11.9%
	600	13.5	10.8	12.8	13.7	13.5	14.0	13.9	13.2	1.14	8.6%
	700	15.2	12.8	15.3	15.0	15.3	15.9	16.1	15.1	1.08	7.2%
	800	11.7	8.6	14.5	13.3	12.8	12.3	11.9	12.1	1.82	15.0%
	900	14.9	12.3	14.6	14.6	15.1	16.1	16.5	14.9	1.35	9.1%
	1000	49.1	9.7	10.8	24.5	38.4	52.3	55.5	34.3	19.42	56.6%
Selectivity [60 s TMPMCT]	100	0.87	0.86	0.89	0.91	0.89	0.87	0.87	0.88	0.014	1.6%
	200	0.84	0.82	0.83	0.86	0.85	0.83	0.82	0.83	0.016	1.9%
	300	0.90	0.87	0.88	0.90	0.89	0.89	0.89	0.89	0.009	1.0%
	400	0.92	0.90	0.91	0.92	0.92	0.92	0.92	0.91	0.007	0.8%
	500	0.95	0.94	0.95	0.96	0.95	0.95	0.95	0.95	0.007	0.7%
	600	0.92	0.93	0.92	0.92	0.92	0.92	0.92	0.92	0.005	0.6%
	700	0.92	0.93	0.92	0.93	0.92	0.92	0.92	0.92	0.005	0.5%
	800	0.95	0.96	0.94	0.94	0.94	0.95	0.95	0.95	0.008	0.8%
	900	0.94	0.95	0.94	0.94	0.94	0.94	0.94	0.94	0.005	0.5%
	1000	0.84	0.96	0.96	0.92	0.87	0.83	0.82	0.88	0.062	7.1%

Selectivity in the case of [40 s TMPMCT and H_2O]

	ALD cycle	Thickness (Å)/ Run number									
		1	2	3	4	5	6	7	avg	std	unif(1σ)
ALD on [40 s TMPMCT with H_2O]	500	10.7	9.9	10.8	10.6	10.5	10.5	11.3	10.6	0.42	4.0%
	600	9.9	9.4	10.1	8.7	8.7	10.1	10.4	9.6	0.68	7.1%
	700	8.7	8.6	8.7	7.8	7.9	9.1	9.2	8.6	0.55	6.4%
	800	8.5	8.0	8.0	8.4	8.1	8.6	9.0	8.4	0.35	4.2%
	900	8.5	7.9	8.0	8.8	8.1	8.4	9.0	8.4	0.41	4.9%
	1000	7.5	6.8	7.1	7.6	7.0	7.4	7.6	7.3	0.31	4.3%
Selectivity [40 s TMPMCT with H_2O]	500	0.93	0.93	0.92	0.93	0.93	0.93	0.92	0.93	0.002	0.3%
	600	0.94	0.94	0.94	0.95	0.95	0.94	0.94	0.94	0.004	0.4%
	700	0.96	0.95	0.96	0.96	0.96	0.95	0.95	0.96	0.003	0.3%
	800	0.96	0.96	0.96	0.96	0.96	0.96	0.96	0.96	0.001	0.1%
	900	0.97	0.97	0.97	0.97	0.97	0.97	0.96	0.97	0.001	0.1%
	1000	0.97	0.97	0.97	0.97	0.98	0.97	0.97	0.97	0.001	0.1%

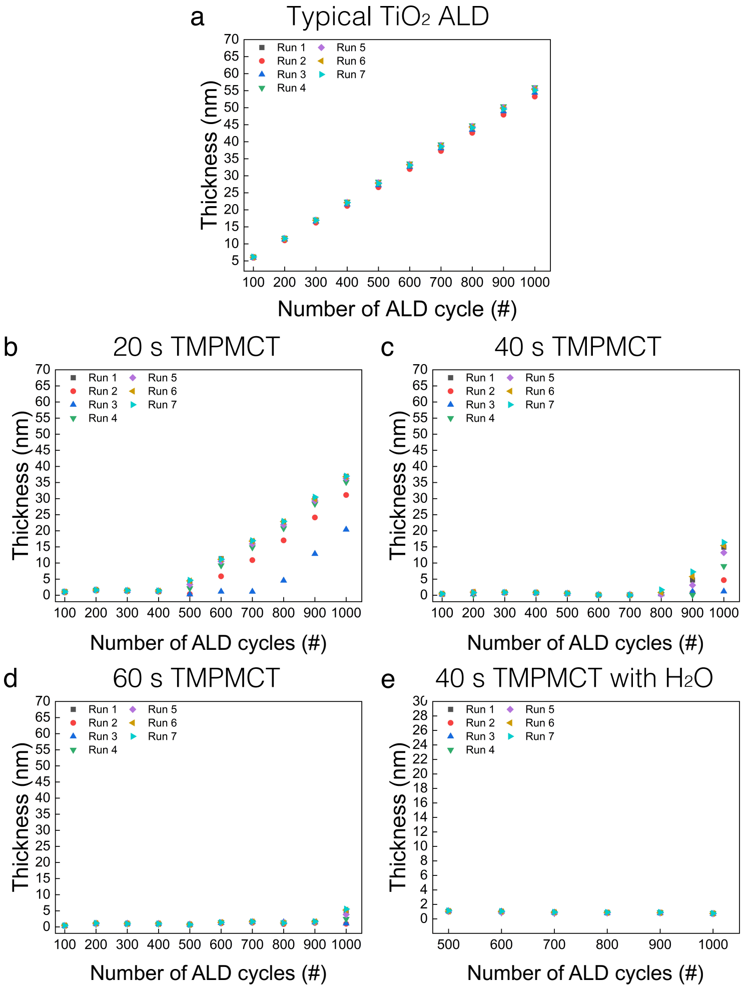

Supplementary Fig. 14: Plots using raw data in Supplementary Table 4. Source data are provided as a Source Data file.

Supplementary References

1. Haider, A., Yilmaz, M., Deminskyi, P., Eren, H. & Biyikli, N. Nanoscale selective area atomic layer deposition of TiO_2 using e-beam patterned polymers. *RSC Adv.* **6**, 106109–106119 (2016).
2. Pattison, T. G. *et al.* Surface Initiated Polymer Thin Films for the Area Selective Deposition and Etching of Metal Oxides. *ACS Nano* **14**, 4276–4288 (2020).
3. Parsons, G. N. Functional model for analysis of ALD nucleation and quantification of area-selective deposition. *Journal of Vacuum Science & Technology A* **37**, 020911 (2019).
4. Avrami, M. Kinetics of Phase Change. II Transformation‐Time Relations for Random Distribution of Nuclei. *J. Chem. Phys.* **8**, 212–224 (1940).
5. Lee, H.-B.-R., Mullings, M. N., Jiang, X., Clemens, B. M. & Bent, S. F. Nucleation-Controlled Growth of Nanoparticles by Atomic Layer Deposition. *Chem. Mater.* **24**, 4051–4059 (2012).
6. Gordon, R. G., Hausmann, D., Kim, E. & Shepard, J. A Kinetic Model for Step Coverage by Atomic Layer Deposition in Narrow Holes or Trenches. *Chem. Vap. Deposition* **9**, 73–78 (2003).
7. Gu, B. *et al.* Computational Modeling of Physical Surface Reactions of Precursors in Atomic Layer Deposition by Monte Carlo Simulations on a Home Desktop Computer. *Chem. Mater.* acs.chemmater.2c00854 (2022) doi:10.1021/acs.chemmater.2c00854.
8. Smith, T. J. MOLView: A program for analyzing and displaying atomic structures on the Macintosh personal computer. *Journal of Molecular Graphics* **13**, 122–125 (1995).
9. Aragón-Muriel, A. *et al.* Synthesis, biological evaluation and model membrane studies on metal complexes containing aromatic N,O-chelate ligands. *Heliyon* **6**, e04126 (2020).

10. Hu, M., Hauger, T. C., Olsen, B. C., Luber, E. J. & Buriak, J. M. UV-Initiated Si–S, Si–Se, and Si–Te Bond Formation on Si(111): Coverage, Mechanism, and Electronics. *J. Phys. Chem. C* **122**, 13803–13814 (2018).
